# Fgf and Wnt signaling interaction in the mesenchymal niche regulates the murine hair cycle clock

**DOI:** 10.1038/s41467-020-18643-x

**Published:** 2020-10-09

**Authors:** Sarina Harshuk-Shabso, Hila Dressler, Christof Niehrs, Emil Aamar, David Enshell-Seijffers

**Affiliations:** 1 The Laboratory of Developmental Biology, The Azrieli Faculty of Medicine, Bar-Ilan university, Safed, Israel; 2 Division of Molecular Embryology, DKFZ-ZMBH Alliance, Deutsches Krebsforschungszentrum (DKFZ), 69120 Heidelberg, Germany; 3 Institute of Molecular Biology (IMB), 55128 Mainz, Germany

**Keywords:** Cell signalling, Developmental biology, Stem-cell niche, Skin stem cells

## Abstract

Tissue growth in the adult is an orchestrated process that often requires biological clocks to time stem cell and progenitor activity. Here, we employed the hair follicle, which cycles between growth and regression in a timely-restricted mode, to show that some components of the hair cycle clock reside within the mesenchymal niche of the hair follicle, the dermal papilla (DP), and both Fgf and Wnt signaling pathways interact within the DP to regulate the expression of these components that include Wnt agonists (*Rspondins*) and antagonists (*Dkk2* and *Notum*). The levels of Wnt agonists and antagonists in the DP are progressively reduced and elevated during the growth phase, respectively. Consequently, Wnt signaling activity in the overlying epithelial progenitor cells decreases, resulting in the induction of the regression phase. Remarkably, DP properties allow Wnt activity in the DP to persist despite the Wnt-inhibiting milieu and consequently synchronize the induction and progression of the regression phase. This study provides insight into the importance of signaling crosstalk in coupling progenitors and their niche to regulate tissue growth.

## Abstract (web summary)

The underlying mechanisms regulating the mouse hair cycle remain poorly understood. Here, the authors find that Fgf and Wnt signaling pathways interact in the mesenchymal niche of the hair follicle to regulate the molecular clock that dictates the duration of hair growth.

## Introduction

Tissue growth and regeneration in the adult often require the activity of specialized stem cell and progenitor populations, but also need to be constrained within a time frame to allow the formation of proper size, shape, and structure^1,2^. Furthermore, tissue removal may also accompany tissue growth to achieve normal function^3^. The hair follicle periodically cycles between growth and regression in a timely coordinated mode^4–6^, providing a powerful model system to explore the molecular mechanisms that time stem cell activity and progenitor behavior.

The mature hair follicle during the growth phase (anagen) of the hair cycle is largely composed of concentric layers of differentiated keratinocytes that comprise the outer root sheet (ORS), inner root sheet (IRS) and hair shaft (HS). At the bottom of the follicle, the dermal papilla (DP), a mesenchymal component of the follicle, is embedded in the hair bulb and surrounded by keratinocytes collectively called matrix cells. Matrix keratinocytes in direct contact with the DP behave as progenitor cells of the hair matrix^7^. Asymmetric divisions renew this progenitor cell compartment and persistently provide vertical flux of transit amplifying progeny that undergo a limited number of divisions and differentiate to form the constituents of the IRS and HS. The persistent addition of these new constituents to the base of the inner layers drives the growth of the hair and its emergence through the skin. At the end of the anagen phase, proliferation in the hair matrix ceases and the regression phase (catagen) ensues. During catagen, apoptosis initiates in the matrix around the DP and spreads from the matrix upwards. Consequently, follicles shorten and regress^8,9^. Concomitantly, the DP is withdrawn with the regressing epithelial strand to lie beneath the permanent portion of the follicle during quiescence (telogen).

Numerous models and theories have been proposed during the last five decades to explain the cyclic nature of the hair follicle^10–18^, and in spite of intensive research, the components and the molecular mechanisms that underlie the hair cycle remain poorly understood. Remarkably, the duration of anagen within a specific mouse strain is largely constant and strictly maintained from individual to individual and from cycle to cycle, suggesting the existence of a biological clock that dictates the periodicity of the hair cycle. Previous studies have shown that Fgf signaling may play an important role in regulating the hair cycle clock^19–23^. *Fgf5* knockout mice display abnormally long hairs as a result of prolonged anagen, while Fgf5 administration during mid anagen results in premature induction of catagen^21–23^. Expression analysis of *Fgf5* in the hair follicle revealed that *Fgf5* is expressed during anagen in the lower ORS all the way to the base of the bulb region including matrix cells adjacent to the bottom of the DP^21^. As catagen induction starts when matrix cells undergo apoptosis in the bulb region^8,9^, Fgf5 may act directly on matrix cells to inhibit their proliferation or/and induce apoptosis. Alternatively, Fgf5 may affect the matrix cells indirectly by signaling to the DP^21,24^. As the proximity of *Fgf5*-expressing cells to the DP is limited to only few cells of the lower part of the DP, the direct mechanism by which Fgf5 acts on matrix cells has been considered more likely. However, the mechanism by which Fgf5 acts to regulate the transition from anagen to catagen remains unknown.

In addition to Fgf signaling, canonical Wnt signaling pathway may also play a role in regulating the hair cycle clock. Ablation of beta-catenin in the matrix or DP during mid anagen results in premature induction of catagen^25,26^. While this clearly illustrates that Wnt signaling activity is required in both the matrix and DP to maintain the anagen phase, it remains unclear whether activation of the Wnt signaling pathway in both compartments converges into a single biological process that regulates the duration of anagen, or Wnt signaling transduction in the matrix and DP independently retain the anagen-maintaining activity of each compartment. Interestingly, forced expression of the constitutive active form of beta-catenin specifically in the DP does not alter the hair cycle^27^. This suggests that while Wnt signaling activity in the DP is required to sustain anagen, it is not sufficient to counteract the catagen-inducing signals. In the current study, we show that Fgf and Wnt signaling pathways interact in the DP to regulate the hair cycle clock by orchestrating the expression of Wnt agonists (Rspondins) and antagonists (Dkk2 and Notum).

## Results

### Fgf signaling in the DP modulates components of the hair cycle clock that regulate anagen duration

**Fig. 1:**
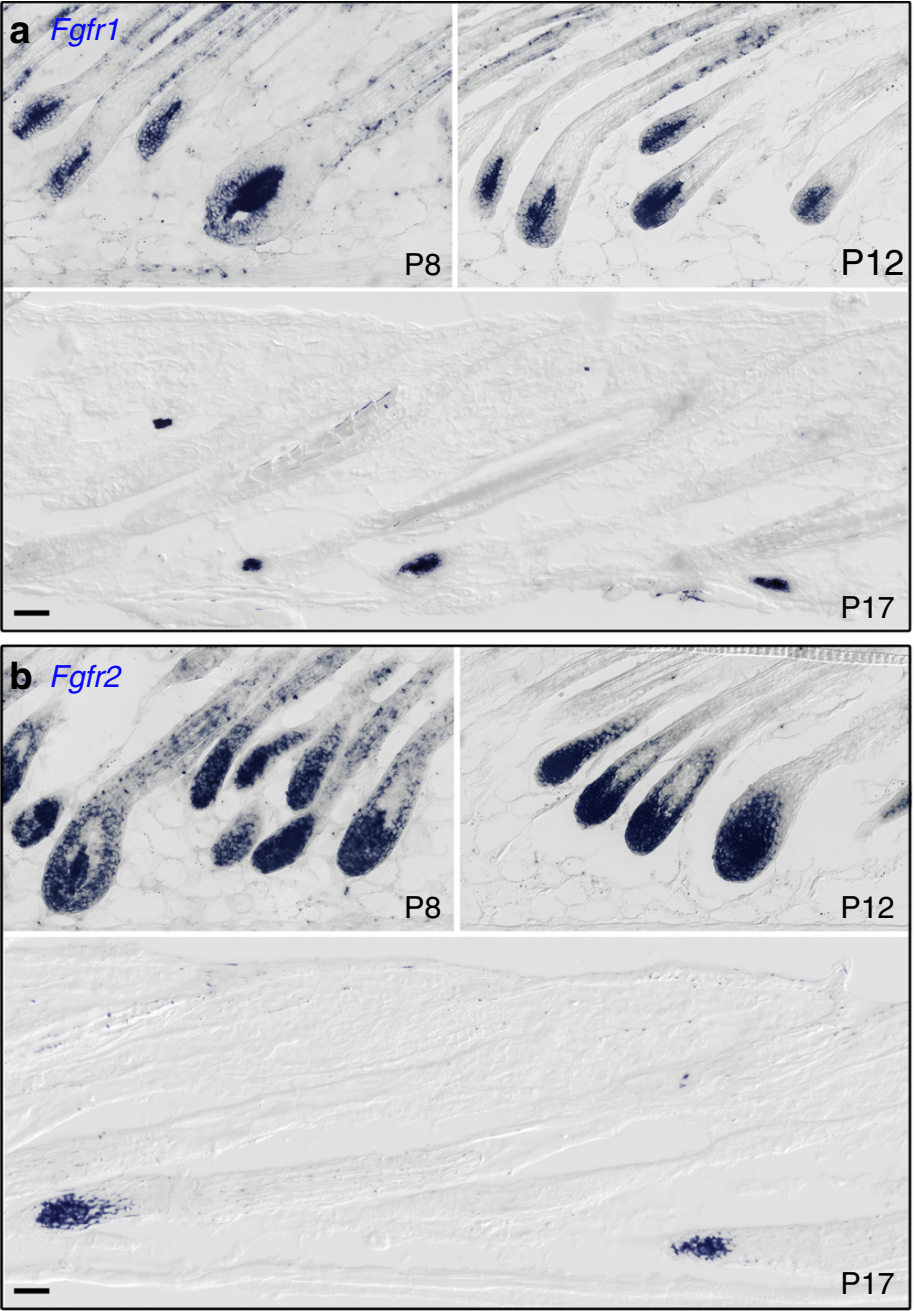
*Fgfr1* and *Fgfr2* are expressed in the DP throughout the anagen and catagen phase. In situ hybridizations for *Fgfr1* (**a**) and *Fgfr2* (**b**) are shown. Note that the transcripts (blue) for both receptors become restricted to the DP during catagen (P17). All images were taken within the same scale and chronologically ordered clockwise. Unpigmented FVB mice were used. *n* > 3 mice per time point. Scale bar, 50 μm.

**Fig. 2:**
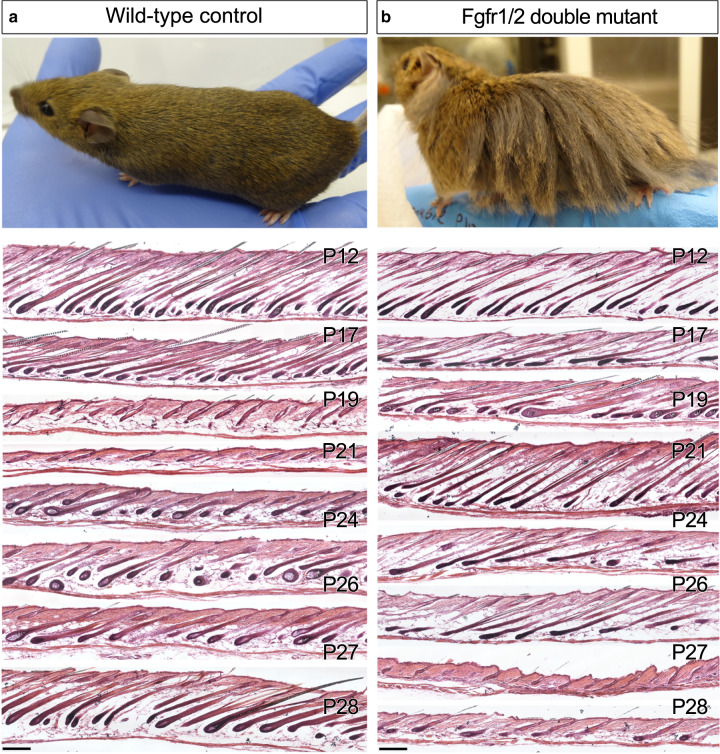
Ablation of Fgf signaling in the DP results in extended anagen. In the upper panels, pictures of a wild type control (**a**) and a Fgfr1/2 double mutant (**b**), taken at postnatal day 50 (P50), are shown (*n* > 10 mice per genotype). The panels below depict HE staining of dorsal skin sections derived from the posterior region of wild-type and mutant mice at different stages of the hair cycle (on the right side, P indicates postnatal day). Note the prolonged anagen in the double mutant. At P19, a wild-type skin section (**a**) is shown to illustrate mid-to-late catagen, while P24 and P26 are examples of Fgfr1/2 double mutant mice (**b**) that are in the transition from anagen to catagen. By contrast, wild-type follicles at P26 (**a**) have already completed regeneration and are in the mid anagen of the second cycle. *n* > 3 mice per genotype per stage. Scale bar, 200 μm.

To unravel the role of Fgf signaling in the DP, we first determined which Fgf receptors mediate Fgf signaling in the DP. In situ hybridization was employed to assess the expression of all four Fgfr receptors during the growth phase. All four receptors were readily detected in the epithelial part of the follicle as previously reported^28^. However, while *Fgfr1* and *Fgfr2* are abundantly expressed in the DP throughout the anagen and catagen phase, only very low levels of *Fgfr3* and *Fgfr4* are detected in this compartment (Fig. 1a, b, Supplementary Fig. 1a–d), suggesting that Fgfr1 and Fgfr2 are the predominant transducers of Fgf signaling in the DP. Subsequently, *Fgfr1* and *Fgfr2* were specifically ablated in the DP during the hair cycle by crossing conditional knockout mouse lines of *Fgfr1* and *Fgfr2* (refs. ^29,30^) with the DP-specific Cor-cre mouse line that expresses the cre recombinase in the DP postnatally^25^. Three genotypically distinct types of mutants were generated; single *Fgfr1*, single *Fgfr2*, and double *Fgfr1/2* mutants (designated dMF1/2). While the hair coat of both *Fgfr1* and *Fgfr2* single mutants appears normal (Supplementary Fig. 2a, b), the double *Fgfr1/2* mutant exhibits extremely long hairs (Fig. 2a, b).

To explore the underlying mechanism of the extended hair length, back skins were harvested from wild-type control and dMF1/2 double mutant mice at different time points during the first and second hair cycles. As the hair cycle gradiently progresses along the anterior–posterior axis, dorsal skins were collected from the posterior region of this axis. Skin sections were used for HE staining to illustrate follicle morphology (Fig. 2a, b). In wild-type mice, catagen is induced around postnatal day 18 (P18), telogen commences at P20, and a new hair cycle is initiated around P22 (Fig. 2a, Supplementary Fig. 3a–e). By contrast, catagen in double mutant mice is initiated between P24 to P26, 6–8 days later than wild type (Fig. 2b). Of note, while catagen induction in wild-type mice in this particular genetic background strictly occurs at P18, catagen induction in the double mutant substantially varies; even at P26, mutant mice are occasionally found in anagen (Fig. 2b). To test whether this extension in anagen is restricted only to the first cycle or also occurs in subsequent cycles, the anagen duration of the second cycle was evaluated. Note that the extended anagen of the first cycle and the large variance in catagen induction of the mutant preclude the direct comparison between control and mutant littermates and thus complicate the analysis. Therefore, the duration of anagen in the second cycle was individually estimated by exploiting the active process of hair pigmentation that occurs from Anagen III to catagen induction (Supplementary Fig. 4). During this period, the skin appears dark, and thus the duration of this period can be visually measured for each individual mouse. As this time frame constitutes most of the anagen phase, its measurement provides a good estimation for the length of anagen. Consistently, the anagen phase is dramatically extended in the absence of Fgf signaling in the DP also during the second cycle (Supplementary Fig. 4). Together these data clearly suggest that some key components of the hair cycle clock that regulate the duration of anagen reside in the DP, and Fgf signaling pathway in the DP modulates the expression and/or activity of these components.

### Profiling the DP transcriptome reveals that two predominant expression programs operate during the growth phase

We hypothesized that at least some of the components that constitute the hair cycle clock are progressively accumulated or degraded in the DP during the anagen phase, and once these molecules surpass a certain threshold, catagen is induced. To test this hypothesis, gene expression profiling of DP cells from control and double mutant mice at multiple time points during anagen of the first cycle was performed by RNA-seq. To isolate DP cells from control and dMF1/2 mutant mice, DP cells were endogenously labeled with YFP by activating the cre-dependent YFP-reporter allele^31^, designed to be included in both control and dMF1/2 mutant mice (Supplementary Fig. 3a–e). Following the dissociation of skin into a single-cell suspension, DP cells were FACS sorted from mice at P10, P12, P14, P16, and P18 (Supplementary Fig. 3f). This spans successive time points during anagen (P10–P16) in both wild-type and mutant mice (Supplementary Fig. 3a–d) and includes a time point during the transition from anagen to catagen (P18) in wild-type mice (Supplementary Fig. 3e). Total RNA was purified from sorted cells and used to prepare sequencing libraries (Supplementary Fig. 3g).

Note that anagen of the first cycle is often considered late follicle morphogenesis, and anagen of subsequent cycles is thought to be the genuine anagen of the hair cycle. While follicle morphogenesis and the anagen phase of the hair cycle were detailedly described and anatomically classified into multiple stages (Stage1–8 and AnaI-VI, respectively)^32,33^, most of these stages refer to early steps of follicle formation (Stage1–7)^32^ or the very early events of anagen during regeneration (AnaI-III) and immediately post regeneration (AnaIV–V)^33^. The last stage of both follicle morphogenesis and the anagen of the hair cycle (Stage8 and AnaVI, respectively) begins when the tip of the HS emerges through the epidermis and lasts most of the growth (anagen) phase of the first and second hair cycles respectively. Furthermore, follicle morphology remains largely unaltered throughout this last and long stage of hair growth. Therefore, Stage8 and AnaVI are often considered equivalent and “monotonous”. Our hypothesis suggests that this “monotonous” stage is molecularly dynamic, and to simplify the analysis, this monotonous stage of anagen during the first cycle, which is the focus of our analysis, was chronologically subclassified into three time frames; early-to-mid anagen (P10–P12), mid-to-late anagen (P14–P16), and late anagen to early catagen (P16–P18).

**Fig. 3:**
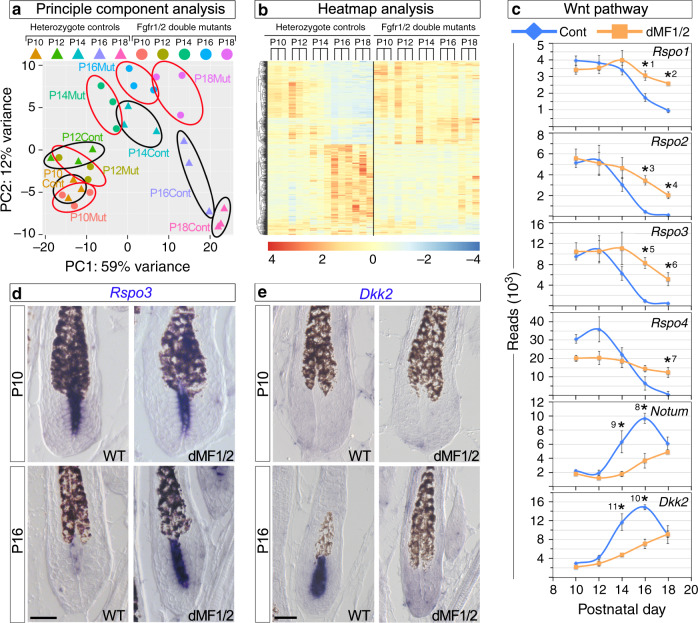
Abrogation of Fgf signaling in the DP results in altered expression of Wnt agonists and antagonists. **a** PCA analysis. Each colored triangle or dot represents the DP transcriptome of a single control or mutant mouse, respectively. Black and red circles demarcate control and mutant mice at a given time point, respectively. *n* = 3 mice per genotype per time point. **b** Heatmap analysis. Two classes of genes were revealed based on their expression pattern: genes whose expression during anagen gradually accumulate in control mice while remains low in the mutant and genes whose expression is progressively reduced in the control while persists at higher levels in mutant mice. **c** Graphic representation of the sequence reads of the RNA-seq analysis for Wnt agonists (*Rspo1–4*) and antagonists (*Notum* and *Dkk2*). Note the different scales of the reads. Data are mean ± SEM. Two-sided modified *t*-test adjusted for multiple comparisons using the Benjamin–Hochberg correction was employed. *1 Padj = 0.001, *2 Padj = 7.14E − 11, *3 Padj = 3.26E − 08, *4 Padj = 3.18E − 57, *5 Padj = 3.48E − 24, *6 Padj = 4.91E − 26, *7 Padj = 4.05E − 38, *8 Padj = 0.0003, *9 Padj = 0.02, *10 Padj = 3.2E − 06, *11 Padj = 4.14E − 07. *n* = 3 mice per genotype per time point. **d**, **e** In situ hybridization of *Rspo3* and *Dkk2* at P10 and P16 is shown to illustrate the specific and dynamic expression of these genes in the DP and their altered expression in the mutant. RNA transcripts are in dark blue and pigmented hair shafts in black. *n* > 5 mice per genotype per time point. Scale bar, 25 μm.

Principle component analysis (Fig. 3a) revealed that the DP transcriptome during early-to-mid anagen (P10–P12) remains relatively unaltered regardless of the genotype. By contrast, the DP transcriptome of wild-type mice during mid anagen to catagen induction (P14–P18) is highly dynamic and progressively undergoes substantial changes. The DP transcriptome of mutant mice during P14–P18 also evolves but is highly distinctive than control. To test the anagen progression hypothesis, we further explored the expression behavior of DP cells during anagen by focusing on genes found to be differentially expressed between control and mutant mice at P16 and analyzing their expression pattern from P10. The focus on this selected set of genes relies on the hypothesis that genes involved in the regulation of catagen induction should be differentially expressed between controls and mutants during late anagen before the initiation of catagen in the control. Samples were chronologically ordered along a timescale and presented as a heatmap for control and mutant mice separately (Fig. 3b). This clearly illustrates the presence of two predominant expression programs in control mice, opposing in their behavior and dynamics. The transcript levels of one program during mid anagen start elevated and progressively decrease during late anagen until they stabilize on certain low levels. Conversely, the expression levels of the other program begin low and gradually incline to its maximum at the transition between anagen and catagen. By contrast, while the same two transcriptional programs do occur in the mutant, the rate of the transcriptional change in both programs is substantially slower. Together, these data are in line with our hypothesis and suggest that the part of the hair cycle clock that orchestrates anagen duration is composed of both accumulating and degrading components.

### The expression of Wnt agonists and antagonists in the DP are progressively altered during anagen in a cooperative manner

KEGG and GO analysis identified numerous signaling pathways that are significantly altered in the mutant DP. Wnt signaling was amongst these pathways. Remarkably, agonists and antagonists of the pathway are combinedly altered in a cooperative mode (Fig. 3c). In wild-type mice, all four *Rspondins*, known to play an important role in augmenting Wnt signaling^34^, progressively decrease during mid-to-late anagen, while the antagonists *Notum* and *Dkk2* gradually accumulate. By contrast, the transcriptional levels of these genes in the mutant initially remain closer to their initial baseline and subsequently are altered in a similar manner to the dynamics of the wild type but at a slower pace. The expression pattern and dynamics of Rspondins, *Dkk2* and *Notum* were further explored in skin sections by in situ hybridization (Fig. 3d, e and Supplementary Fig. 5). All four Rspondins are either predominantly expressed in the DP with substantially lower levels in the matrix (*Rspo1*, *Rspo2*, and *Rspo4*; Supplementary Fig. 5a) or their expression is restricted only to the DP (*Rspo3*; Fig. 3d), suggesting the DP is the major source of Rspondins in the bulb region. Note that while the transcripts of all four Rspondins are readily observed in the DP of P10 mice regardless of the genotype, expression of *Rspo2* and *Rspo3* in the DP of wild-type mice at P16 is reduced beneath the detection levels (Fig. 3d and Supplementary Fig. 5a). Expression of *Rspo1* and *Rspo4* in wild-type mice at P16 is still sufficiently high to be readily detected by in situ hybridization at this stage but become undetectable during catagen (Supplementary Fig. 5a, b). In contrast to wild-type mice, the transcripts of all four Rspondins are abundantly detected in the DP of P16 dMF1/2 mutant mice, consistent with the RNA-seq analysis.

DP-confined expression is also observed for *Notum* and *Dkk2* during late anagen (Fig. 3e, Supplementary Fig. 5c). At P10, the expression levels of *Dkk2* and *Notum* in both wild-type and dMF1/2 mutant mice are too low to be detected by in situ hybridization. At P16, however, the expression of *Dkk2* and *Notum* in wild-type mice dramatically increases to the levels that are readily noticed by in situ hybridization. These high levels of *Dkk2* and *Notum* in the wild-type DP persist through early catagen and decline to undetectable levels during the end of catagen or mid catagen, respectively (Supplementary Figs. 6a and 7a). By contrast, the expression of *Dkk2* and *Notum* in the mutant at P16 remains beneath the detection levels (Fig. 3e and Supplementary Fig. 5c).

The persistent expression of *Dkk2* and *Notum* in wild-type DP during catagen suggests that both play an instructive role in regulating the normal execution of catagen and its progression. Furthermore, their DP expression at P16 may coincide with but not necessarily reflect the regulation of catagen induction. To precisely map whether the expression of *Dkk2* and *Notum* in the DP precedes catagen initiation, both proliferation and apoptosis were evaluated at P16 (Supplementary Fig. 8). During catagen induction, proliferation in matrix cells ceases and subsequently programed cell death is initiated within the matrix in close proximity to the DP^8^. Immunostaining for Ki67 of skin sections from control and dMF1/2 mutant mice at P16 revealed that matrix cells are highly proliferative regardless of the genotype. Furthermore, Tunel staining failed to detect apoptotic cells within the matrix, clearly indicating that wild-type follicles at P16 represent genuine late anagen. Together these data demonstrate that expression of *Dkk2* and *Notum* in the DP is normally elevated prior to catagen induction and thus suggest that both may participate in regulating catagen initiation.

### Persistent expression of Rspo3 in the DP of the dMF1/2 mutant contributes to the extended anagen

The expression pattern and dynamics of the Wnt agonists and antagonists in the DP suggests that Wnt signaling activity in the bulb of wild-type mice decreases during mid-to-late anagen, and this reduction promotes catagen induction. This hypothesis predicts that abrogation of Rspondins specifically in the DP should result in premature induction of catagen. However, the co-expression of all four Rspondins predominantly in the DP is likely to complicate the analysis of a single ablation as a result of functional redundancy or accumulative effects that are proportional to the levels of each Rspondin. Consistently, single ablation of *Rspo3* in the DP does not result in a measurable premature induction of catagen during the first and the second cycle. Note however that based on our RNA-seq and in situ analyses, the most significant changes in gene expression between control and dMF1/2 mutant mice were observed for *Rspo2* and *Rspo3*, and thus, their persistent expression in the DP of the dMF1/2 mutant is likely to predominantly contribute to the extended anagen. To explore this proposition, a triple mutant of *Rspo3*, *Fgfr1* and *Fgfr2* (designated tM/F1/2/R3) was generated and the duration of anagen during the first and second cycle was evaluated. While the prolonged anagen of the double dMF1/2 mutant is comparable to that of the triple tMR3/F1/F2 mutant during the first cycle, anagen duration of the triple mutant during the second cycle shortens relatively to the double mutant but still longer than controls (Supplementary Fig. 4). This suggests that (1) persistent expression of *Rspo3* in the absence of *Fgfr1* and *Fgfr2* in the DP contributes to the extended anagen and (2) Rspondin expression in the DP is required to regulate the duration of anagen.

### The Wnt agonists and antagonists from the DP predominantly act on matrix cells despite their autocrine potential

**Fig. 4:**
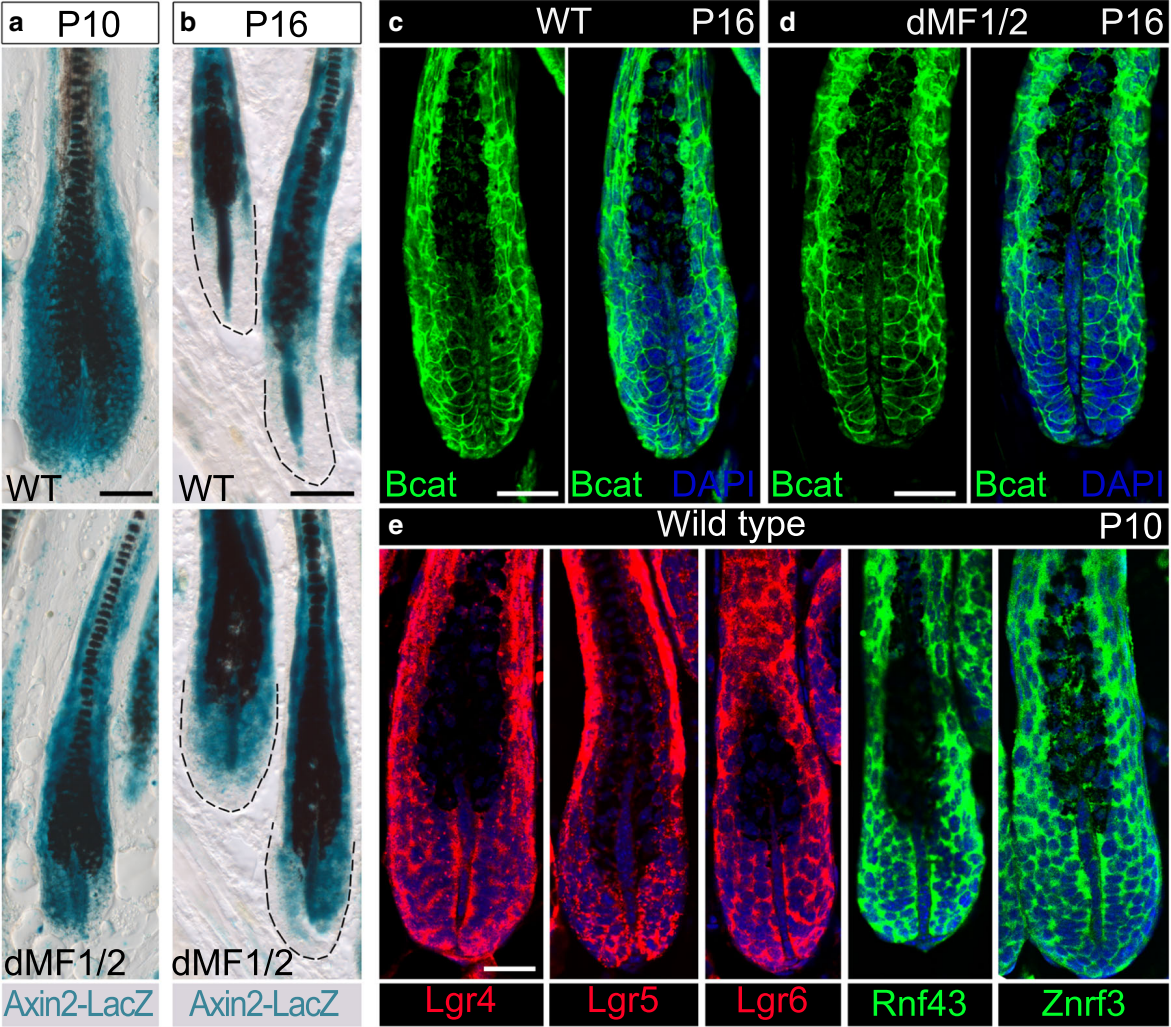
Wnt signaling activity during mid-to-late anagen is progressively decreased only in the matrix to promote catagen induction. **a** X-Gal staining in skin sections from wild-type (upper panel) and Fgfr1/2 double mutant (lower panel) mice shows LacZ expression at P10. Note that Wnt signaling activity is readily detected in both the matrix and DP regardless of the genotype. Also note the comparable signal between the DP and the matrix. *n* = 3 mice per genotype. **b** X-Gal staining during late anagen (P16) revealed that Wnt activity in the wild type (upper panel) is diminished in the matrix but persists in the DP. By contrast, Wnt activity in the mutant (lower panel) persists in both the matrix and DP. Note however the signal in the DP is higher than the matrix. The dashed black line demarcates the boundaries of the follicle. *n* = 5 mice per genotype. **c**, **d** Immunostaining for beta-catenin during late anagen is shown to illustrate the presence of activated beta-catenin in the nuclei of DP cells in both wild-type (**c**) and mutant (**d**) littermates. For each genotype, the same follicle is shown twice: on the left, only beta-catenin fluorescent image is presented, and on the right, a merge of beta-catenin and nuclear staining (DAPI) is displayed. *n* = 6 mice per genotype. **e** Wild-type skin sections from P10 mice were used to immunostain all the components of the machinery required to transduce Rspondin activity. Nuclei are in blue (DAPI). Note that none of these components is detected in the DP. *n* > 3 mice. Scale bar in (**a**, **b**), 50 μm. Scale bar in (**c**–**d**), 25 μm.

To explore the proposition that Wnt signaling activity in the bulb is normally reduced during late anagen, the *Axin2-lacZ* Wnt-reporter allele^35^ was introduced in both control and dMF1/2 mutant mice. Remarkably, the dynamics of Wnt activity in the matrix differ substantially than those in the DP during anagen of wild-type mice. Wnt signaling activity in wild-type matrix is reduced at P16 as compared to early anagen of wild-type mice at P10, while Wnt activity in wild-type DP during late anagen persists and even intensifies (Fig. 4a, b, upper panels). Furthermore, the reduction of Wnt activity in wild-type matrix during mid-to-late anagen occurs progressively (Supplementary Fig. 9). While the intensification of Wnt activity in the DP also occurs in the dMF1/2 mutant, Wnt activity in the matrix of mutant mice at P16 remains high and comparable to P10 (Fig. 4a, b, lower panels). Immunostaining for beta-catenin at P16 revealed activated beta-catenin localized to the nuclei of DP cells in both wild-type and mutant mice (Fig. 4c, d), corroborating the persistent activity of Wnt signaling specifically in the DP during late anagen. Together these data suggest that DP cells are resistant to the activity of Rspondins, *Dkk2* and *Notum*, and matrix cells are the cellular target for these agonists and antagonists.

Note that while Notum is a secreted enzyme that hydrolyzes the palmiteoylate adducts from Wnt ligands and thus inactivates the ability of Wnts to bind Fzd receptors^36^, Rspondins and Dkk2 activities require a whole molecular machinery within the target cells. We hypothesized that the confined action of these Wnt agonists and antagonists on matrix cells is predominantly achieved by restricting the expression of the molecular machinery, required to mediate the activity of Rspondins and Dkk2, to matrix cells. To explore this hypothesis, the expression pattern of the molecular machinery required to mediate the activity of Rspondins and Dkk2 was assessed in wild-type mice (Fig. 4e, Supplementary Figs. 10 and 11). Znrf3 and Rnf43 are transmembrane E3 ubiquitin ligases that remove Wnt receptors from the membrane by targeting the Fzd receptors for degradation^37,38^. Rspondins bind both the E3 ubiquitin ligase and a member of Lgr family (Lgr4, Lgr5, and Lgr6) to form a ternary structure that abolishes the ubiquitination activity of the E3 ligase and thus augments Wnt activity by allowing surface accumulation of Fzd receptors. Immunostaining and in situ hybridization for Lgr4, Lgr5, Lgr6, Znrf3, and Rnf43 revealed that all are abundantly expressed in the epithelial part of the follicle including the matrix but are not detected in the DP (Fig. 4e, Supplementary Fig. 10). Dkk2 inhibits Wnt signaling by binding to both the Wnt co-receptor Lrp5/6 and the transmembrane receptor Krm1/2, which results in internalization of Lrp5/6 and consequently reduction in the ability of the cell to respond to Wnt ligands^39^. In situ hybridization for both *Krm1* and *Krm2* revealed that both are undetectable in the DP but highly abundant in the epithelial part of the follicle (Supplementary Fig. 11). Of note, while *Krm1* is widely expressed in the follicular epithelium, *Krm2* is asymmetrically localized to a small group of matrix cells adjacent to the DP. Together these data suggest that only matrix cells are appropriately equipped to respond to the activity of Rspondins and Dkk2 and provide molecular insight to the reduction in Wnt signaling activity only in the matrix during late anagen.

**Fig. 5:**
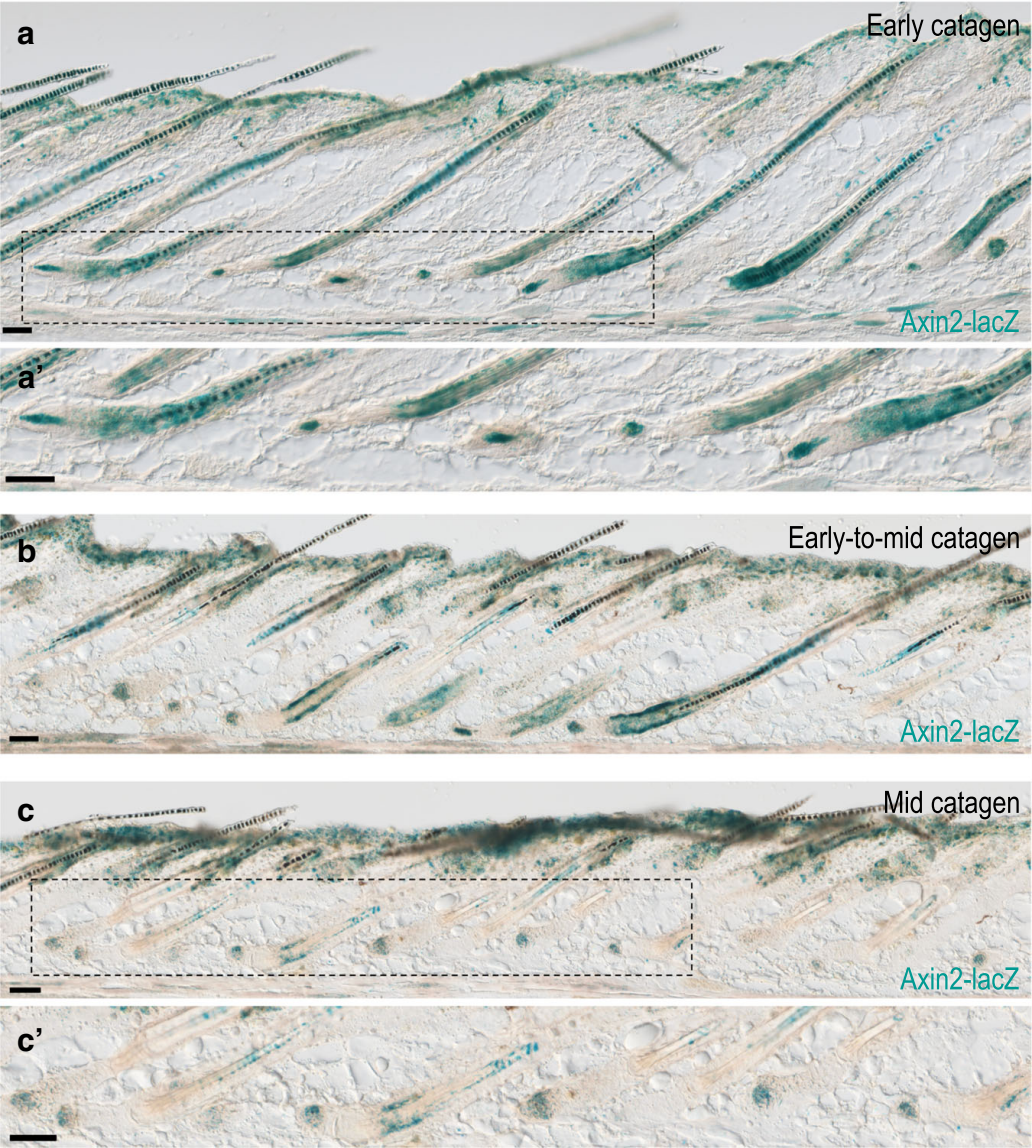
Wnt signaling activity during catagen. LacZ staining of dorsal skin sections from adjacent regions along the anterior–posterior axis of a wild-type mouse at P18 are shown. **a**, **a**′ Posterior dorsal region is displayed to illustrate the high levels of Wnt activity in the DP and the lack of Wnt activity in the epithelial part of the follicle adjacent to the DP during early catagen. In (**a**′), a higher magnification of the field demarcated by the dashed rectangle in (**a**) is presented. *n* = 3 mice. **b** Middle dorsal region is shown to demonstrate Wnt activity during early-to-mid catagen. *n* = 3 mice. **c**, **c**′ Anterior region is depicted to show Wnt activity during mid catagen. In (**c**′), a higher magnification of the field outlined by the dashed rectangle in (**c**) is shown. *n* = 3 mice. Scale bar, 50 μm.

The persistent expression of *Dkk2* and *Notum* during catagen suggests that inhibition of Wnt activity within the epithelial part of the follicle may be required also for catagen progression, and the apparent resistance of the DP to the inhibition activity of these Wnt antagonists suggests that Wnt signaling activity in the DP is retained during catagen. The *Axin2-lacZ* Wnt-reporter allele^35^ was further used to follow Wnt activity during catagen. Consistently, Wnt activity within the lower part of the regressing follicle was observed only within the DP and is reduced during mid-to-late catagen (Fig. 5a–c).

### Wnt signaling in the DP is required for Wnt activity in the matrix and is stimulated by Wnt ligands from the matrix

**Fig. 6:**
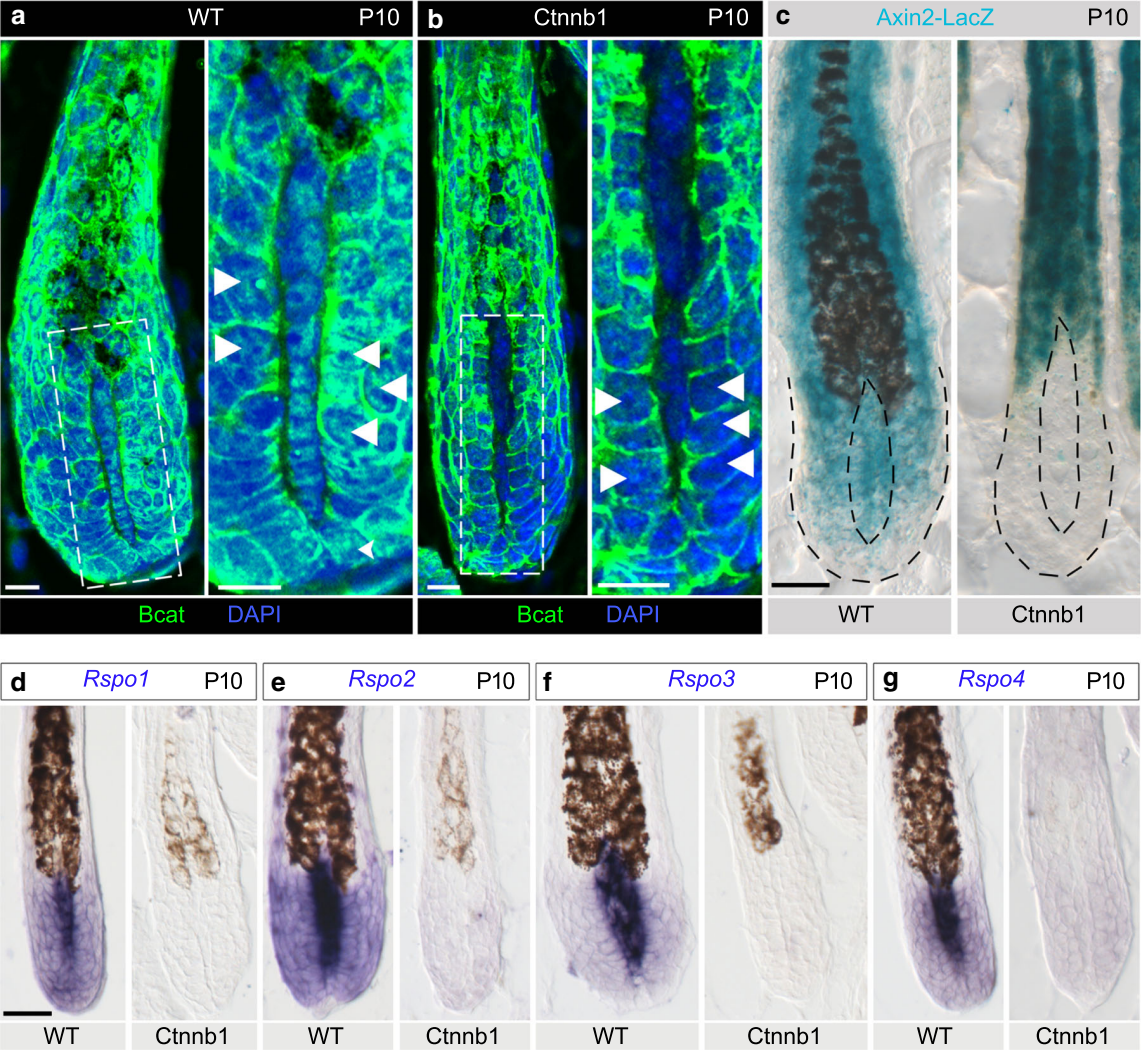
Ablation of beta-catenin specifically in the DP results in reduction of Wnt signaling activity in the matrix. **a** Immunostaining for beta-catenin during mid anagen (P10) of a wild-type follicle is displayed. Nuclei are in blue (DAPI). A higher magnification of the field outlined with the dashed white square in the left panel is presented on the right panel. Note that in addition to the membrane localization of beta-catenin in matrix cells, activated beta-catenin is readily detected also in the nuclei of these cells (white arrowheads). *n* = 3 mice. **b** beta-catenin mutant mice were used to immunostain for beta-catenin at P10 to illustrate the efficient deletion of beta-catenin in the DP and the dramatic reduction of nuclear beta-catenin in matrix cells (white arrowheads). A higher magnification of the field outlined with the dashed white square in the left panel is shown on the right panel. *n* = 3 mice. **c** X-Gal staining in skin sections from P10 wild-type (left panel) and mutant (right panel) littermates demonstrates that Wnt activity in the mutant is abolished in both the DP and matrix. The internal dashed black line outlines the DP and the external black dashed line demarcates the follicle boundaries. *n* = 3 mice per genotype. **d**–**g** In situ hybridization for Rspondins on skin sections from P10 wild-type and mutant littermates illustrates the absence of Rspondin transcripts in both the DP and matrix of the mutant. *n* = 3 mice per genotype. Scale bar in (**a**, **b**), 10 μm. Scale bar in (**c**–**g**), 25 μm.

The reduction of Wnt signaling in the matrix during late anagen (Fig. 4) is consistent with the observation that ablation of beta-catenin in the matrix results in premature induction of catagen^26^ and suggests that this reduction is the physiological mechanism to induce catagen. However, ablation of beta-catenin in the DP also results in premature induction of catagen^25^. We hypothesized that Wnt signaling in the matrix directly maintains the anagen phase while Wnt activity in the DP indirectly regulates the duration of anagen by modulating Wnt signaling in the matrix. To explore this hypothesis, the gene for beta-catenin (*Ctnnb1*) was specifically ablated in the DP by crossing the Cor-cre line with a mouse line that harbors a floxed allele of beta-catenin as previously described^25,27^. In line with these prior reports, catagen in the Ctnnb1 mutant is prematurely induced at P12, 4 days earlier than wild type (Supplementary Fig. 12a–e). Note that catagen induction in wild-type mice of this genetic background occurs at P16 (ref. ^25^). Immunostaining for beta-catenin at P10 when both wild-type and Ctnnb1 mutant follicles are in anagen confirmed the efficient deletion of beta-catenin specifically in the DP of the Ctnnb1 mutant (Fig. 6a, b). Furthermore, in addition to its membrane localization, beta-catenin in wild-type mice was readily detected also in the nuclei of matrix cells, a clear indication of Wnt signaling activity in these cells. Intriguingly, nuclear localization of beta-catenin in the matrix of Ctnnb1 mutant mice is reduced, suggesting that Wnt signaling activity in the matrix is decreased as a consequence of beta-catenin ablation in the DP. The *Axin2-LacZ* Wnt-reporter allele was further used to corroborate that Wnt signaling activity is abolished in both the DP and the matrix when beta-catenin was ablated in the DP (Fig. 6c) and supported that Wnt activity in the matrix depends on Wnt activity in the DP.

Previous reports have shown that during mid-to-late anagen Wnt ligands are expressed in the matrix and are not detected in the DP^40–42^, suggesting the matrix is the only functional source of Wnt ligands in the bulb region that autocrinally and paracrinally activate Wnt signaling in the matrix and DP, respectively. To test this hypothesis and to exclude the possibility of undetectable but functional expression of Wnt ligands in the DP during late anagen, the ability of DP cells to secrete Wnt ligands was curtailed by ablation of the *Wls* gene specifically in the DP. For this, a mouse line that harbors a floxed allele of *Wls*^43^ was crossed with the Cor-cre mouse line. Such genetic manipulation neither altered the first hair cycle nor affected the HS, corroborating that the matrix is the predominant functional source of Wnt ligands in the bulb region.

### Wnt signaling activity in the DP activates the expression of Rspondins and is essential for the extended anagen in the dMF1/2 mutant

**Fig. 7:**
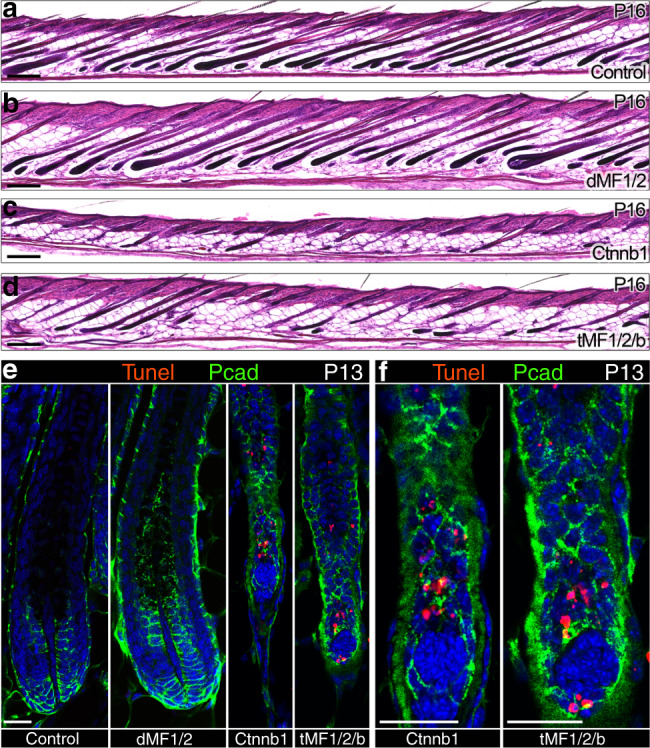
Concomitant ablation of Fgfr1, Fgfr2, and beta-catenin specifically in the DP results in premature induction of catagen. **a**–**d** HE staining of skin sections from control, dMF1/2, Ctnnb1, and tMF1/2/b mice at P16 is shown. Note the unsynchronized nature of catagen in both Ctnnb1 and tMF1/2/b mutant mice. *n* > 3 mice per genotype. **e** Tunel staining of P13 skin sections from control, dMF1/2 double mutant, Ctnnb1 mutant, and tMF1/2/b triple mutant is shown to illustrate the normal apoptosis around the DP during the premature catagen. Pcad immunostaining was also included to outline the DP. Nuclei are in blue (DAPI). *n* = 3 mice per genotype. **f** Higher magnifications of the fields surrounding the DP of Ctnnb1 and tMF1/2/b mutant follicles in e are displayed. Scale bar in (**a**–**d**), 200 μm. Scale bar in (**e**, **f**), 25 μm.

Previous reports demonstrated that Rspondins in some settings are transcriptional targets of Wnt signaling^44^. This suggests that Wnt activity in the DP regulates Wnt signaling in the matrix by activating the expression of Rspondins in the DP. Indeed, in situ hybridization of skin sections from P10 wild-type and Ctnnb1 mutant littermates unveiled not only that expression of all Rspondins in the DP is abolished, but also the low expression of Rspondins in the matrix is diminished in Ctnnb1 mutant mice (Fig. 6d–g). This is consistent with Rspondins being target genes of Wnt signaling and with the dramatic reduction in Wnt activity in the matrix when beta-catenin is ablated in the DP. Furthermore, this suggests that the extended anagen, observed when *Fgfr1* and *Fgfr2* were ablated in the DP, requires the presence of active Wnt signaling in the DP. To test this proposition, a triple mutant of *Ctnnb1*, *Fgfr1*, and *Fgfr2* (designated tMF1/2/b) was generated. Consistently, follicles within the triple mutant enter prematurely to catagen similar to a single Ctnnb1 mutant (Fig. 7a–f), corroborating the functional interaction between Fgf and Wnt signaling pathways in the DP.

### Wnt signaling activity in the DP activates the expression of Dkk2 and Notum in a timely dependent manner

The absence of Fgf signaling in the DP of the dMF1/2 mutant at P16 prevents the elevation of both *Dkk2* and *Notum*, suggesting that *Dkk2* and *Notum* are transcriptionally activated by Fgf signaling in the DP. However, *Dkk2* and *Notum* were previously shown to be target genes of Wnt signaling as part of a negative feedback loop^45–47^. To explore whether *Dkk2* and *Notum* are target genes of Wnt signaling in the DP, in situ hybridization was performed to compare their expression between wild-type and Ctnnb1 mutant littermates. We first tested the hypothesis that Wnt activity in the DP suppresses the expression of *Dkk2* and *Notum*, while Fgf signaling in the DP activates the expression of *Dkk2* and *Notum* by derepressing the activity of Wnt signaling. This predicts dramatic elevation of *Dkk2* and *Notum* levels in the DP of Ctnnb1 mutant. However, at P10, both *Dkk2* and *Notum* are undetectable regardless of the genotype (Supplementary Fig. 13a, b), refuting the hypothesis that Wnt activity in the DP suppresses the expression of *Dkk2* and *Notum*. At P12, when catagen is induced in the Ctnnb1 mutant, *Dkk2* and *Notum* remain undetectable, and this lack of expression persists throughout the premature catagen phase of the Ctnnb1 mutant (Supplementary Fig. 13a, b), in contrast to the normal expression of these genes and the restricted activity of Wnt signaling in the DP during catagen of wild-type mice (Fig. 5, Supplementary Figs. 6a and 7a). This suggests that *Dkk2* and *Notum* are transcriptionally activated in the DP by Wnt signaling. Of note, this activation occurs only during late anagen despite the presence of Wnt activity at earlier times. The latter and the observation that Wnt activity in the DP is intensified during late anagen suggest that activation of *Dkk2* and *Notum* requires high levels of Wnt signaling activity, or alternatively, a longer period of Wnt signaling reception is required to activate the expression of these genes. Furthermore, together these data support that (1) while both Dkk2 and Notum may participate in regulating the initiation of catagen, they are not required to induce catagen; and (2) the reduction of Rspondin levels during mid-to-late anagen is the preponderant driving force that promotes and induces catagen.

### Catagen induction and progression in the dMF1/2 mutant are apparently normal

The dMF1/2 mutant follicles remain longer in anagen but eventually enter catagen. To explore whether the molecular clock that induces catagen in wild-type mice also operates in the double mutant and therefore catagen initiation and progression in the double mutant are normal but only delayed, in situ hybridization for Rspondins, *Dkk2* and *Notum* was performed during the late anagen and catagen of the double mutant. At P22, the expression of *Rspo2*, *Rspo3*, and *Rspo4* in mutant DP is almost completely abolished, and this extinct expression persists during the catagen phase of the mutant (Supplementary Fig. 14a). The transcript levels of *Rspo1* in the mutant remain high and are readily detected at P22 but become undetectable during catagen. Furthermore, immunostaining for Ki67 and Tunel labeling at P22 revealed that mutant follicles at this stage still sustain the proliferative state of the matrix, demonstrating that P22 in the double mutant is a genuinely late anagen stage (Supplementary Fig. 8). Together these data suggest that reduction in Rspondin expression in the DP during mid-to-late anagen is the driving force that induces catagen also in the double mutant. In contrast to Rspondins, the expression levels of *Dkk2* and *Notum* in the double mutant are elevated during late anagen at P22 similar to control mice at P16 (Fig. 3e, Supplementary Figs. 5c and 14a). Note however, while the drop of *Dkk2* during catagen in the dMF1/2 mutant is similar to that of wild-type mice (Supplementary Fig. 6), the drop of *Notum* in the dMF1/2 mutant appears to occur earlier as the levels of *Notum* in the DP of the dMF1/2 mutant during catagen is consistently lower than control mice (Supplementary Fig. 7). Together these data suggest that catagen induction and progression in the dMF1/2 double mutant are largely normal. This is further corroborated by the observation that Wnt activity during late anagen to mid catagen of the dMF1/2 mutant becomes restricted to the DP and is comparable to that of late anagen to mid catagen of control mice (Fig. 5, Supplementary Fig. 14a, b).

In the absence of *Fgfr1* and *Fgfr2* in the DP, expression of Rspondins is maintained. However, this maintenance lasts for a certain period, and eventually, Rspondin expression is extinguished to allow catagen induction, suggesting the involvement of additional mechanisms in regulating the suppression of Rspondins. While the transcripts of *Fgfr3* and *Fgfr4* are barely detected in the DP of wild-type mice throughout the anagen phase (Supplementary Fig. 1c, d), the expression of these genes may be upregulated in the double mutant during late anagen to compensate for the loss of *Fgfr1* and *Fgfr2*. To test this hypothesis, in situ hybridization for *Fgfr3* and *Fgfr4* was performed on skin sections from controls at P16 and dMF1/2 mutants at P22 (Supplementary Fig. 15a, b). Regardless of the genotype, while the transcripts of *Fgfr3* and *Fgfr4* were abundantly observed in the matrix, very low levels were detected in the DP, refuting the compensation hypothesis and suggesting that additional tyrosine receptor kinases and/or signaling pathways are involved in regulating the duration of anagen and catagen induction.

## Discussion

**Fig. 8:**
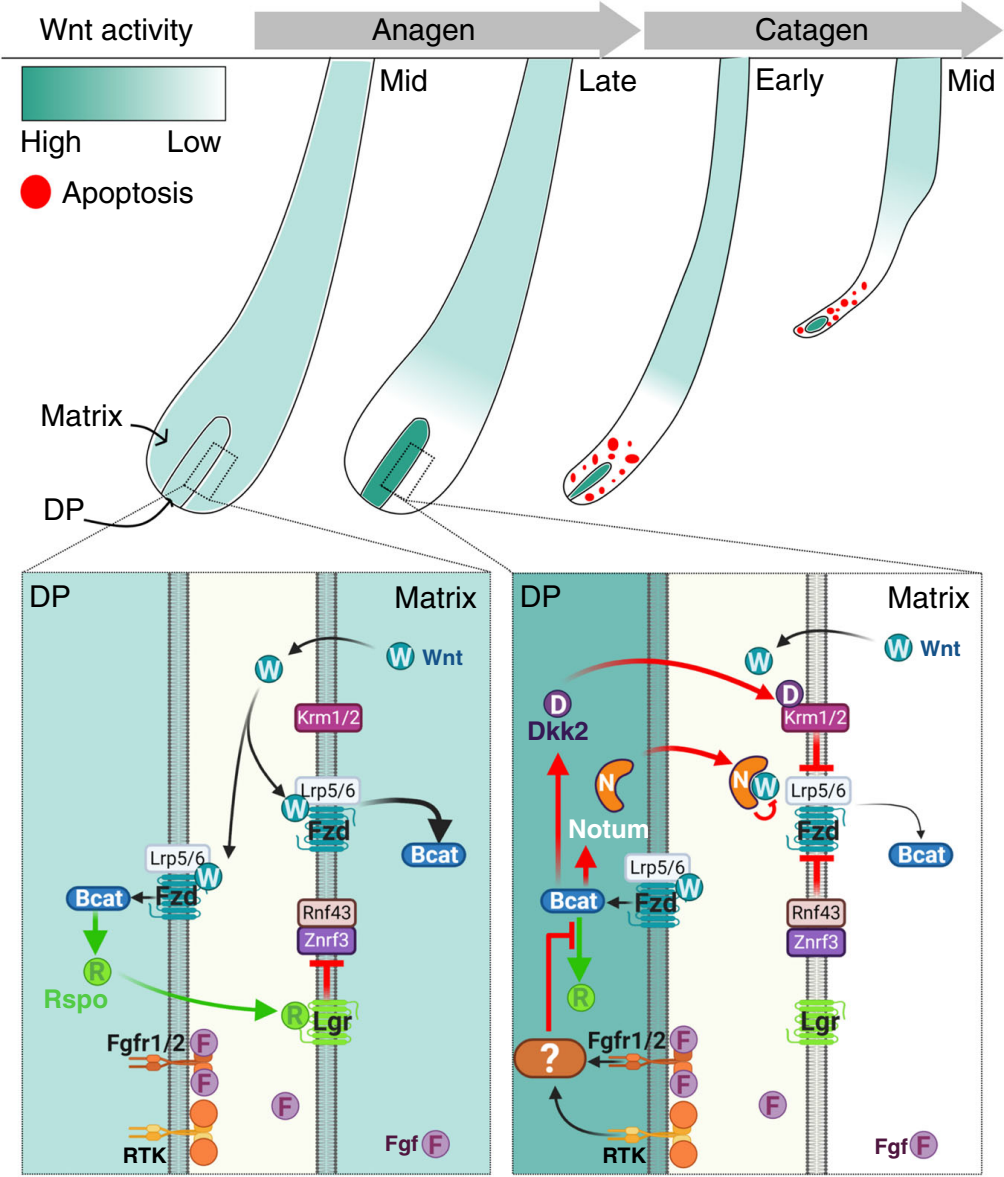
Graphical summary of the positive and negative feedback loops that regulate the duration of anagen. Wnt ligands from the matrix activate Wnt signaling in both matrix and DP cells. Wnt activity in the DP induces the expression of Rspo that act on matrix cells to augment Wnt signaling by suppressing the inhibitory action of Znrf3/Rnf43 on Fzd receptors. This positive feedback loop maintains anagen. Fgf signaling in the DP uncoils this positive loop by repressing Rspo and consequently induces catagen. Concomitantly to the reduction in Rspo expression, a negative feedback loop is activated by Wnt activity in the DP that upregulates the Wnt antagonists Dkk2 and Notum. The inhibitory action of these antagonists further dampens Wnt signaling in the matrix to synchronize catagen induction. Note that the absence and presence of multiple Wnt inhibitory pathways in the DP and matrix respectively allow Wnt activity in the DP to be uncoupled from Wnt activity in the matrix and thus enable the simultaneous incline and decline of Wnt activity in the DP and matrix, respectively. This illustration is created with BioRender.com.

Hair follicles undergo cycles of growth (anagen), regression (catagen), quiescence (telogen), and regeneration. In each phase of the cycle, follicles adopt a different morphology and structure, and consequently, the transitions between these phases involve dramatic morphological alterations that require not only the presence of epithelial stem and progenitor cell populations but also entail the coordination of multiple biological processes. Interestingly, such complexity is orchestrated within relatively precise time frames, suggesting that the hair follicle possesses a biological clock that dictates the periodicity of the hair cycle. Numerous models and theories have been proposed during the last five decades to explain the cyclic nature of the hair follicle^10–17^, and the hair cycle as a clock was previously postulated on the ground of systems biology approach^17,18^. In the current study, we revealed that the crosstalk between Fgf and Wnt signaling pathways in the DP generates adjustable positive and negative feedback loops that molecularly couple the epithelial and mesenchymal compartments in order to regulate the duration of anagen. Furthermore, the distinct properties of matrix and DP cells uncouple Wnt activity in the DP from Wnt activity in the matrix and thus allow the synchronization of catagen induction. At the mechanistic level, this type of control is consistent with the concept of the hair cycle clock, and a graphical summary of suggested model based on the data of the current study is shown in Fig. 8.

A previous work demonstrated that Wnt signaling activity in the matrix maintains the anagen phase^26^, suggesting that reduction of Wnt signaling activity in the matrix to a certain threshold is the underlying mechanism to physiologically induce catagen. Consistently, our study illustrated that such decline of Wnt activity in the matrix does occur during late anagen, and further demonstrated that Wnt signaling in the matrix depends on Wnt activity in the DP. This dependency stems from (1) the ability of Wnt signaling in the DP to activate the expression of all four Rspondins, (2) the predominant expression of Rspondins in the DP, and (3) the intrinsic features of matrix cells that require Rspondins to augment their susceptibility to Wnt ligand activity (Fig. 8). This allows the DP to regulate the duration of anagen. Furthermore, Wnt ligands during mid-to-late anagen are largely expressed in the matrix and not in the DP^40–42^, suggesting that the matrix is the predominant source of Wnt ligands that activate the pathway in both the DP and the matrix. Specific ablation of *Wls* in the DP, performed in the current study, corroborated this proposition. Together, these create a unique positive feedback loop that intertwines the Wnt signaling pathway within the epithelial–mesenchymal interactions to regulate Wnt activity in the matrix and thus control the duration of the growth phase.

Fgf signaling in the DP modulates this positive loop by progressively suppressing the expression of Rspondins (Fig. 8). The kinetics by which this reduction occurs define the period required to reduce Wnt activity in the matrix and to promote the induction of the regression phase, and consequently, control the duration of anagen. Remarkably, this decline in expression of Rspondins in wild-type mice starts ~4 days before the appearance of any morphological signs of catagen induction. This contradicts the current paradigm that strictly distinguishes between anagen and catagen and suggests that catagen at the molecular level is induced during mid-to-late anagen. Note that suppression of Rspondins by Fgf signaling in the DP occurs in the presence of active Wnt signaling in DP cells (Figs. 3d and 4b–d, Supplementary Fig. 5a), suggesting that Fgf signaling downregulates the expression of Rspondins downstream to beta-catenin activation. This is in line with a previous report illustrating that forced expression of the constitutive active form of beta-catenin specifically in the DP does not alter the hair cycle^27^. Mechanistically, Fgf signaling in the DP may antagonize beta-catenin activity in the DP at the promoter level of Rspondins, predicting a wide range of promotor activity that depends on the particular promoter. This is corroborated by the observation that ablation of beta-catenin in the DP results in complete abolishment of all Rspondins at the transcript level (Fig. 6d–g), while abrogation of Fgf signaling in the DP preferentially affects the expression of Rspo2 and Rspo3 (Fig. 3d, Supplementary Fig. 5a).

It is noteworthy that while ablation of *Fgfr1* and *Fgfr2* in the DP results in anagen extension, mutant follicles eventually enter catagen. Furthermore, the reduction in Rspondins expression in the DP of the Fgfr1/2 double mutant does occur during the extended anagen but apparently at a slower pace, corroborating that catagen induction at the molecular level occurs long before the actual regression of the follicle and suggesting that additional molecular components or pathways are involved in regulating the same molecular pacemaker. While elevation of *Fgfr3* and *Fgfr4* in the DP of the dMF1/2 mutant during the extended anagen to compensate for the loss of Fgfr1 and Fgfr2 was not observed, the very low levels of *Fgfr3* and *Fgfr4* may be sufficient for contributing to the regulation of the hair cycle clock in the DP but require a longer time to suppress the expression of Rspondins. Alternatively, other receptor tyrosine kinases are also involved in regulating the hair cycle clock in the DP through cooperative activation of the same intracellular signal transduction pathways activated by Fgfr, and ablation of Fgfr receptors in the DP reduces the activation levels of these pathways but does not abolish them completely.

Concomitant to the decline in Rspondins expression, the Wnt antagonists *Dkk2* and *Notum* in the DP are elevated during late anagen and persist at least throughout early-to-mid catagen, suggesting a cooperative mode of action between agonists and antagonists to regulate catagen induction (Fig. 8). However, ablation of beta-catenin specifically in the DP results in premature induction of catagen despite the lack of elevation in both *Dkk2* and *Notum*. This suggests not only that *Dkk2* and *Notum* are target genes of Wnt signaling in the DP but also that *Dkk2* and *Notum* are dispensable for catagen induction, raising the question of what is the precise role of these antagonists in catagen induction and progression. In addition to premature induction of catagen, catagen induction and progression are extremely unsynchronized in the absence of beta-catenin in the DP despite the apparently normal apoptosis observed in the regressing epithelial strand^25^. This supports the notion that Wnt signaling activity in the DP is also required to synchronize catagen induction and progression in addition to its role in the maintenance of the anagen phase. We hypothesize that this function of Wnt signaling activity is mediated by activating the expression of *Dkk2* and *Notum* in the DP during late anagen to overcome fluctuations between follicles in the pace by which Wnt activity in the matrix is reduced and thus robustly coordinates catagen induction. Furthermore, the current analysis revealed that Wnt activity in the DP operates by orchestrating both positive and negative feedback loops. While the positive loop preserves Wnt activity in the matrix to maintain anagen, the negative loop assures that differences in the rate by which Wnt signaling activity in the overlying matrix decreases during late anagen are overrode to synchronize catagen induction.

The suppression of Rspondins by Fgf signaling and the activation of Wnt antagonists by Wnt signaling only during late anagen and not at earlier times when both pathways are active suggest that signaling activation per se is not sufficient to repress or activate the expression of these genes, respectively. Instead, the duration of signaling reception may also contribute to these functions of Fgf and Wnt signaling in the DP. Alternatively, signaling levels must surpass a certain high threshold to repress or induce these genes. Unfortunately, sensitive methods to directly evaluate Fgf signaling levels in the DP are lacking and therefore preclude us from experimentally testing the latter hypothesis for Fgf signaling. By contrast, the observation that Wnt signaling activity is elevated specifically in the DP during mid-to-late anagen is consistent with this hypothesis. Future studies that manipulate the levels of both signaling pathways along a continuum are required to directly explore these hypotheses.

While the current study did not aim to address the regulation of Fgf signaling in the DP upstream to the Fgf receptors, previous works suggest that *Fgf5* is likely to contribute to this regulation. *Fgf5* is expressed in the lower ORS and lower matrix^21^. Furthermore, *Fgf5* knockout mice exhibit extended anagen. Combined with our analysis, these data together suggest that Fgf5 acts on DP cells to regulate the duration of anagen. Alternatively, Fgf5 function is relayed to the DP through activation of other Fgf ligands in the matrix that trigger Fgf transduction in the DP. However, anagen in the *Fgf5* knockout mice is extended by 3 days^22^ while ablation of *Fgfr1* and *Fgfr2* in the DP results in anagen extension between 6 and 8 days. This suggests that a combined action of a few Fgf ligands is involved. Consistently, several Fgf ligands are known to be expressed during anagen in both the epithelial compartment and the DP^28^. Future functional analysis of these ligands will decipher their role in this process and dissect their relative contribution.

## Methods

### Mice

Fgfr1Flox/Flox (Fgfr1^tm5Sor^)^29^, Fgfr2Flox/Flox (Fgfr2^tm1Dor^)^30^, Ctnnb1Flox/Flox (Ctnnb1^tm2Kem^)^48^, ROSA26 YFP reporter (Gt(ROSA)^26S*ortm*1C*os*^)^31^, and Axin2-lacZ Wnt-reporter (Axin2^tm1Wbm^)^35^ mouse lines were obtained from Jackson Lab. The Rspo3Flox/Flox line^49^ was previously generated by Christof Niehrs laboratory. The DP-specific Corin-cre (Corin^tm2Bamo^) mouse was previously generated^25^ and kindly provided by Bruce Morgan (Harvard medical school).

Crosses for the Fgfr analysis were designed to obtain the following genotypes:

Controls: [*Cor-cre/+; Fgfr1Flox/+; Fgfr2Flox/+*] OR [+*/*+*; Fgfr1Flox/Flox; Fgfr2Flox/Flox*]

Double mutants (dMF1/2): [*Cor-cre/+; Fgfr1Flox/Flox; Fgfr2Flox/Flox*]

Crosses for the beta-catenin analysis were designed to obtain the following genotypes:

Controls: [*+/+; Ctnnb1Flox/Flox*]

Mutants: [*Cor-cre/+; Ctnnb1Flox/Flox*]

Crosses for the triple mutant (tMF1/2/b) analysis were designed to obtain the following genotypes:

Controls: [*+/+; Fgfr1Flox/Flox; Fgfr2Flox/Flox; Ctnnb1Flox/+*] OR [+*/*+*; Fgfr1Flox/Flox; Fgfr2Flox/Flox; Ctnnb1*+*/+*]

Double mutants: [*Cor-cre/+; Fgfr1Flox/Flox; Fgfr2Flox/Flox; Ctnnb1Flox/+*] OR [*Cor-cre/+;Fgfr1Flox/Flox;Fgfr2Flox/Flox;Ctnnb1*+*/+*]

Triple mutants: [*Cor-cre/+; Fgfr1Flox/Flox; Fgfr2Flox/Flox; Ctnnb1Flox/Flox*]

Crosses for the triple mutant (tMF1/2/R3) analysis were designed to obtain the following genotypes:

Controls: [*+/+; Fgfr1Flox/Flox; Fgfr2Flox/Flox; Rspo3Flox/+*] OR [+*/*+*; Fgfr1Flox/Flox; Fgfr2Flox/Flox; Rspo3*+*/+*]

Double mutants: [*Cor-cre/+; Fgfr1Flox/Flox; Fgfr2Flox/Flox; Rspo3Flox/+*] OR [*Cor-cre/+; Fgfr1Flox/Flox; Fgfr2Flox/Flox; Rspo3*+*/+*]

Triple mutants: [*Cor-cre/+; Fgfr1Flox/Flox; Fgfr2Flox/Flox; Rspo3Flox/Flox*]

The Federation of Laboratory Animal Science Associations guidelines were followed to house mouse colonies and experimental animals. Animals were maintained and bred in a temperature-controlled room. Food and water were available ad libitum, and a cycle of 12 h light/dark was employed. The Institutional Animal Care and Use Committee of Bar Ilan University approved the experimental protocols.

### Hair cycle variation and skin collection

Anagen duration varies between mouse lines and strains. In most lines used in this study, catagen of the first cycle in wild-type and control mice is induced around P16. This includes all lines with the conditional allele of beta-catenin and the FVB strain. In the genetic background of the Fgfr1/2 double mutant, however, catagen of the first cycle in wild-type and control mice is induced around P18.

Dorsal skins for the analysis of follicle morphology by HE staining shown in Fig. 2 were harvested from the posterior region. For the lacZ staining shown in Fig. 5, adjacent dorsal regions along the anterior–posterior axis from the same mouse were harvested. For all the rest of the study, middle dorsal region along the anterior–posterior axis was used.

### Histology and immunofluorescence

For immunostaining, dorsal skins were collected, fixed for 16 h at 4 °C in 4% paraformaldehyde (PFA)/PBS, serially dehydrated in sucrose (10–15–20%), embedded in optimal cutting temperature (OCT) compound, frozen on liquid nitrogen, and cryosectioned (7–10 µm). Sections were fixed in ice-cold acetone/methanol (1:1) for 5 min. Subsequently, antigen retrieval was performed by boiling the sections for 5 min in citrate buffer, pH 6 in a microwave. Sections were then cooled to room temperature, permeabilized in methanol for 10 min, washed for 10 min three times in PBS and blocked in 10% heat inactivated sheep serum (HISS) in PBS for 2 h. Sections were incubated overnight at 4 °C with primary antibodies (Abs), washed three times for 10 min in PBS, and incubated with secondary Abs for 1 h at room temperature. Sections were then washed three times for 10 min in PBS and mounted with DAPI FluoroMount-G (Electron microscopy sciences).

To analyze morphology, hematoxylin and eosin (HE) was utilized to stain fixed sections using standard methods.

Primary Abs: Rabbit anti-Lgr4 (1:100, Abcam Ab224480), Rabbit anti-Lgr5 (1:100, Abcam Ab75850), Rabbit anti-Lgr6 (1:100, Abcam Ab214325), Rabbit anti-Rnf43 (1:100, Abcam Ab217787), Rabbit anti-Znrf3 (1:100, Bioss Abs, 9141-R), mouse anti-Bcat (1:100, Abcam Ab6301), Rat anti-Pcad antibody (1:100; BD, #MAB761), Rabbit anti-Ki67 (1:100; Abcam Ab15580).

Secondary Abs: Donkey anti-Rabbit FITC-conjugated, 1:1000 (Jackson ImmunoResearch cat#711-095-152), Donkey anti-Rabbit TRITC-conjugated, 1:1000 (Jackson ImmunoResearch cat#711-025-152), Donkey anti-mouse AlexaFluor488-conjugated, 1:500 (Jackson ImmunoResearch, cat#715-545-150).

### In situ hybridization

Fixed sections from dorsal skins were used for nonradioactive in situ hybridization with Dig labeled RNA probes corresponding to nucleotides (nts) 1127–1689 of Fgfr1 (GenBank Acc. No. NM_010206), nts 1690–2105 of Fgfr2 (NM_010207), nts 2082–2586 of Fgfr3 (NM_001163215), nts 469–869 of Fgfr4 (NM_469-869), nts 958–1395 of Lgr4 (NM_172671), nts 1227–1823 of Lgr5 (NM_010195), nts 1018–1423 of Lgr6 (NM_001033409), nts 720–1313 of Rnf43 (NM_172448), nts 509–773 of Znrf3 (NM_001080924), nts 717–1212 of Rspo1 (NM_138683), nts 1033–1469 of Rspo2 (NM_001357957), nts 921–1348 of Rspo3 (NM_028351), nts 300–760 of Rspo4 (NM_001040689), nts 1168–1481 of Notum (NM_175263), nts 998–1503 of Dkk2 (NM_020265), nts 522–1040 of Krm1 (NM_032396), nts 193–619 of Krm2 (NM_028416).

For signal detection, BM purple substrate (Roche) was used.

### X-gal staining

For X-Gal staining, fresh frozen dorsal skins were used. To keep the harvested skin flat while embedding in OCT compound, skins were stretched on a membrane and immediately frozen on liquid nitrogen. 20-µm cryosections were fixed for 10 min in 0.2% glutaraldehyde (Sigma Aldrich), washed three times in PBS for 5 min, and incubated overnight at 37 °C with 1 mg/ml X-gal (Sigma Aldrich). Sections were washed in PBS three times for 5 min and mounted with Immu-Mount mounting medium (Thermo scientific).

### Tunel staining and co-immunostaining for Pcad

10-µm skin sections were washed twice with PBS, fixed with 4% PFA for 10 min and again washed twice with PBS-0.1% Tween. Subsequently, sections were incubated with ProtK (10 µg/ml) for 5 min and washed twice with PBS-0.1% Tween. For Tunel staining, the “In situ Cell Death Detection Kit-TMR red” (Roche) kit was used according to the manufacturer’s instructions. Briefly, 50 µl Enzyme solution and 550 µl Label solution were mixed and applied on the sections, incubated for 60 min at 37 °C in a humidified box in the dark. Slides were washed three times in PBS then mounted with DAPI. When co-stained for P-Cadherin, the ProtK step was omitted, and after the last three washes in PBS, slides were blocked for 2 h in 10% HISS in PBS. Sections were overnight incubated at 4 °C with Rat anti-Pcad antibody (1:100; BD, #MAB761), washed in PBS three times for 10 min, and incubated for 1 h at room temperature with Donkey anti-Rat Cy5 antibody (1:500; Jackson immunoresearch, #712-175-753). Sections were then washed in PBS three times for 10 min and mounted with DAPI FluoroMount-G (Electron microscopy sciences).

### Microscopy

Imaging of in situ hybridization and X-gal staining was performed with Zeiss upright AxioImagerM2 through a 20× objective with tiling mode using Zen Blue 2.3 software. HE staining was imaged with Zeiss upright AxioImagerM2 or Zeiss slide scannerZ1 through a 20× objective. For immunofluorescence, Zeiss LSM780 inverted confocal microscope was used to acquire images through a 20× objective using the Zen Black 11 (service pack 7) software. Adobe Photoshop CS5.1 was employed to process all images.

### Cell sorting

Single-cell suspension from whole skin was obtained by placing the dermis side down in 0.25% Trypsin (GIBCO) at 4 °C overnight, minced and stirred in 0.2% collagenase for 1 h at 37 °C. Strainers (100, 70, and 40 µM) were used to serially filter the dissociated cells. YFP-positive cells were twice FACS sorted on MoFlo Astrios (Backman Coulter): enrichment 1–2 mode was applied for the first sort, and purify 1 mode was employed for the subsequent sort. FACS analyses were performed using Summit program.

### RNA sequencing

FACS-sorted cells were utilized to purify total RNA using the RNeasy Plus Micro kit (QIAGEN) according to manufacturer’s instructions. RNA integrity was tested using the Agilent RNA Pico Kit and Bioanalyzer at the Genome Technology center of the Faculty of Medicine Bar Ilan University. In total, 100 ng of total RNA were used to deplete ribosomal RNA, and the Nebnext Ultra Directional RNA kit (NEB, #E7420L) was utilized to generate libraries for Illumina sequencing. The dsDNA HS Assay Kit and QUBIT (Molecular Probes, Life Technologies) were employed to quantify the sequencing libraries. Additional measurements to test the quality of the sequencing libraries were performed by qPCR analysis using the illumina P7 and P5 primers. A standard library was used for optimal load on the Illumina HiSeq 2500 instrument. 2 nM of the library was denatured in 0.1 M NaOH for 5 min at room temperature. In total, 10 pM was loaded onto the Flow Cell with 1% Phix library control and sequenced using a 61-cycles single-read sequencing mode.

### Bioinformatics

Trimmomatic was applied to trim and quality filter the 61-base single-end reads, and then Tophat (version 2) was utilized to map the reads to the mouse genome (NCBI38/mm10). Mapped reads for each annotated ENSEMBL gene (GRCm38.p4) were counted using HTSeq-count tool. DESeq2 was used to perform read count normalization and differential gene expression analysis.

### Statistics and reproducibility

Statistical methods to predetermine sample size were not used. Sample size and biological replicates are indicated in the figure legends and at least three biological replicates per genotype were used. Data are presented as mean ± SEM. A Padj value of <0.05 was considered significant.

### Reporting summary

Further information on research design is available in the Nature Research Reporting Summary linked to this article.

## Supplementary information

Supplementary Information

Reporting Summary

## Data Availability

Upon request, all the data that support the results of this study are available from the corresponding author. The RNA-seq dataset is available in the GEO repository (GSE155837). Source data are provided with this paper.

## Source data

Source Data
