# Supplementary material for: Fgf and Wnt signaling interaction in the mesenchymal niche regulates the murine hair cycle clock
Source: Nat Commun. 2020 Oct 9;11:5114. doi: 10.1038/s41467-020-18643-x (PMC7547083; doi:10.1038/s41467-020-18643-x)
Supplement: Supplementary file 1 — Supplementary Information [file 41467_2020_18643_MOESM1_ESM.pdf]

SUPPLEMENTARY INFORMATION

Fgf and Wnt signaling interaction in the mesenchymal niche regulates the murine hair cycle clock

Sarina Harshuk-Shabso[1], Hila Dressler[1], Christof Niehrs[2,3], Emil Aamar[1], and David Enshell-Seijffers[1]

1 The Laboratory of Developmental Biology, The Azrieli Faculty of Medicine, Bar Ilan university, 8 Henrietta Szold, Safed, Israel

2 Division of Molecular Embryology, DKFZ-ZMBH Alliance, Deutsches Krebsforschungszentrum (DKFZ), 69120 Heidelberg, Germany

3 Institute of Molecular Biology (IMB), 55128 Mainz, Germany

Correspondence to: david.enshell@biu.ac.il

This PDF file includes:

Supplementary figures 1 to 15

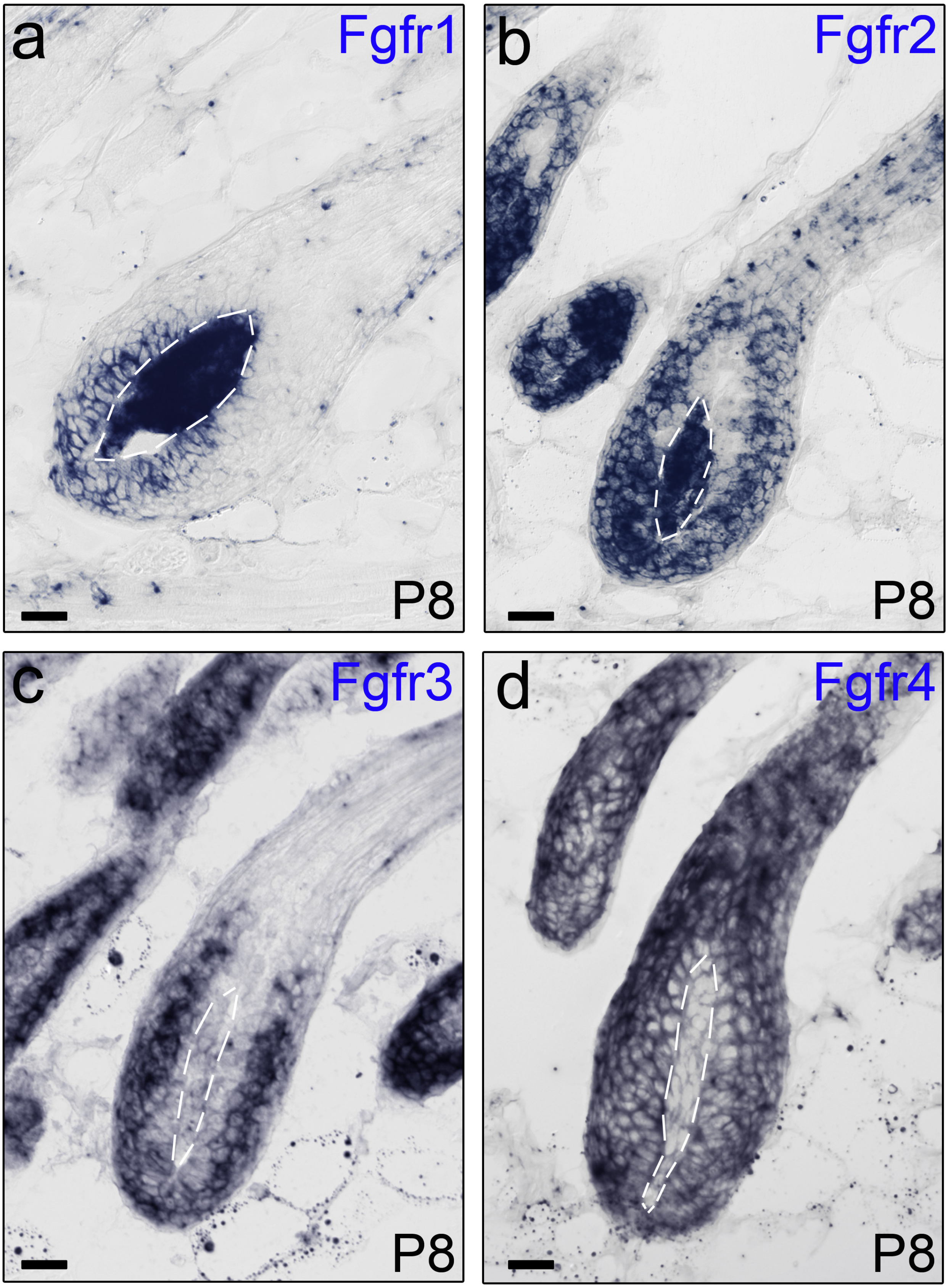

Supplementary Figure 1. *Fgfr1* and *Fgfr2* are abundantly expressed in the DP during the anagen phase. In-situ hybridization for all four Fgfr receptors during the early anagen of the first hair cycle (P8). Note the abundant expression (dark blue) of *Fgfr1* (a) and *Fgfr2* (b) and the very low expression of *Fgfr3* (c) and *Fgfr4* (d) in the DP. Skins from wild type albino FVB mice at P8 were used. White dashed line outlines the DP. n>3 mice. Scale bar, 25 μm.

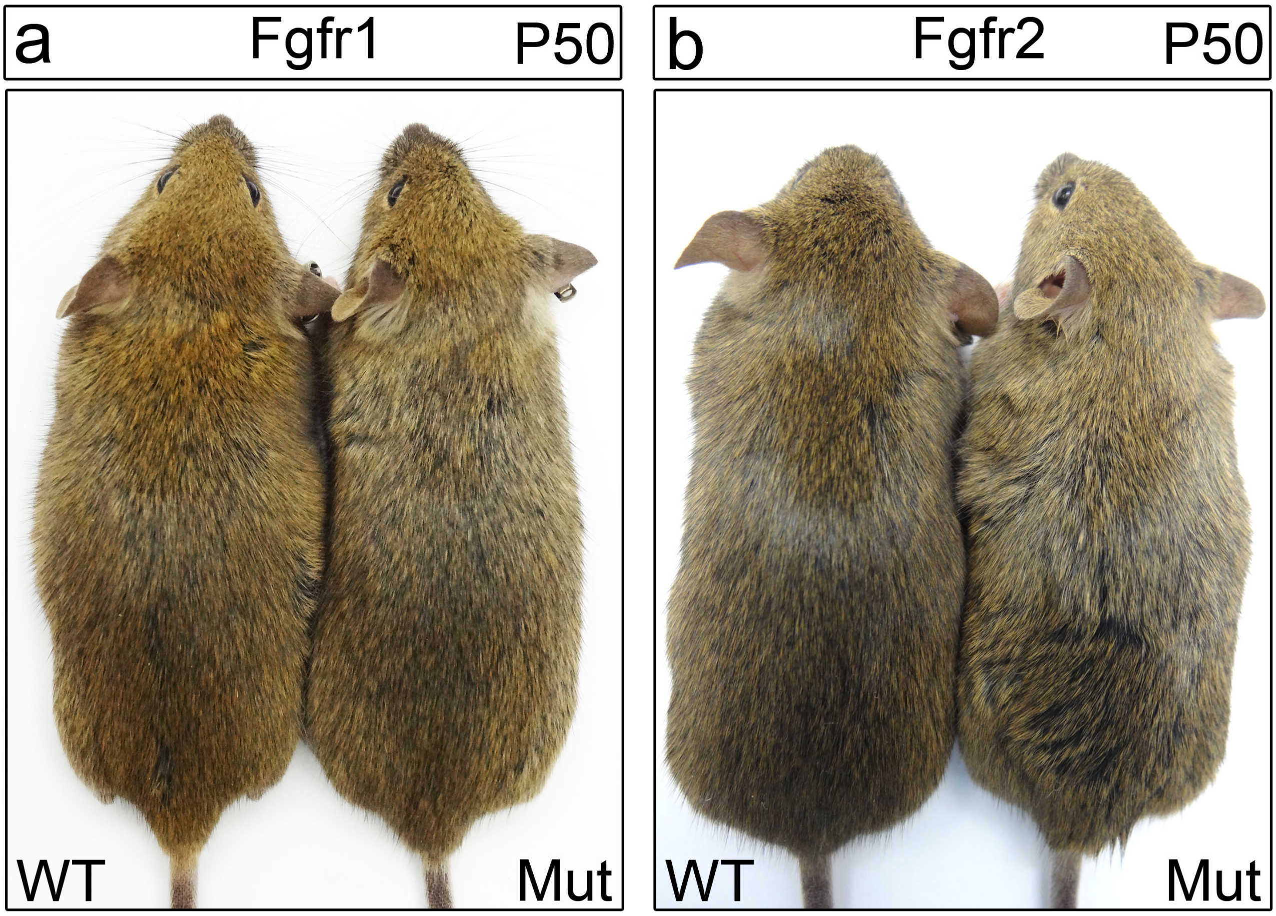

Supplementary Figure 2. *Fgfr1* and *Fgfr2* play redundant roles in the DP. Single ablation of *Fgfr1* (a) or *Fgfr2* (b) specifically in the DP does not result in an observable hair phenotype. Pictures were taken at P50 following two hair cycles. n>10 mice per genotype.

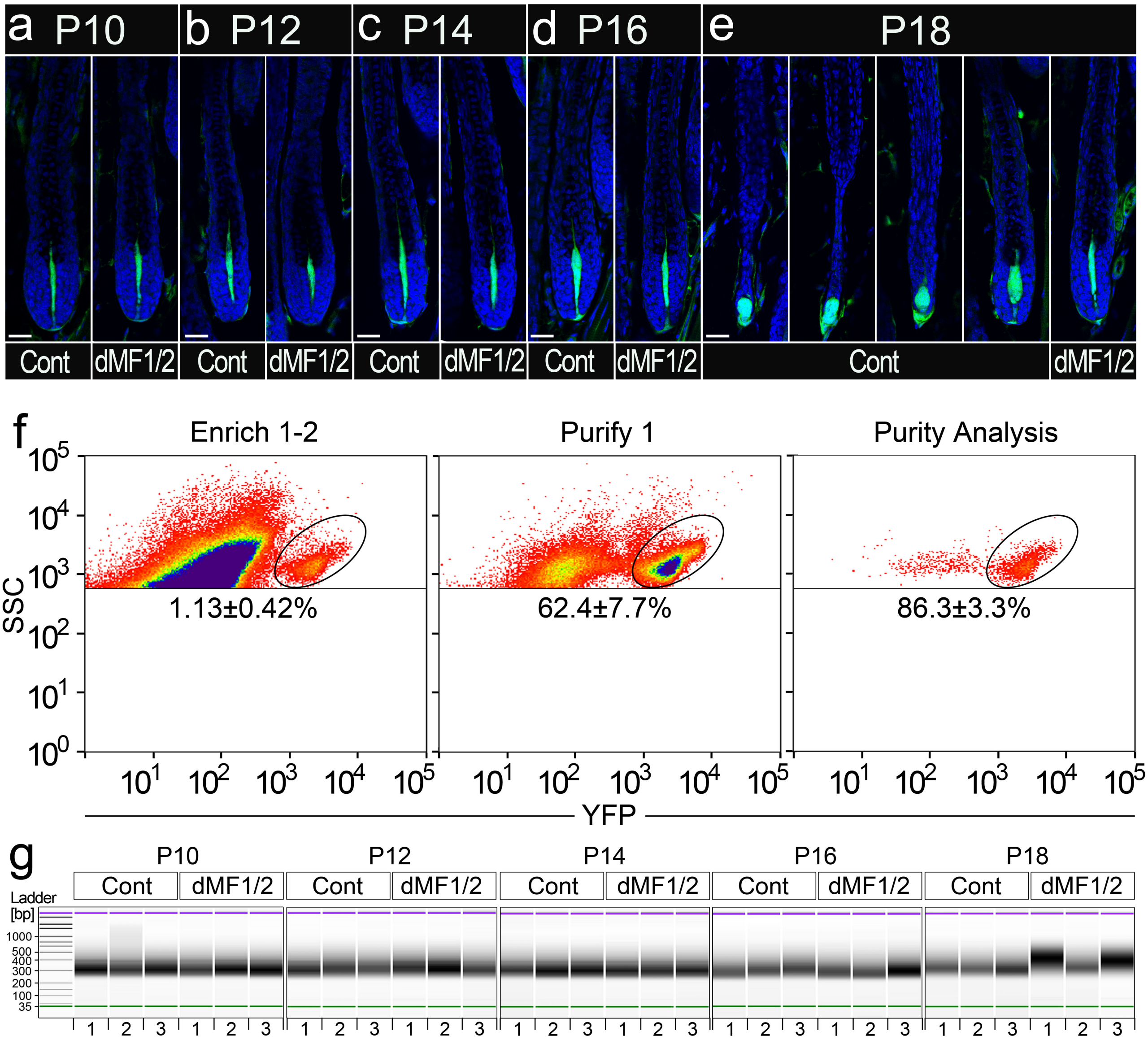

Supplementary Figure 3. FACS cell-sorting methodology to isolate DP cells for RNA-seq. (a-e) To endogenously label DP cells with YFP, the Rosa26-YFP reporter allele was introduced into both control and dMF1/2 double mutant mice. In a-d, a single follicle from control (left panel) and dMF1/2 mutant (right panel) littermates is shown to illustrate the YFP (green) expression specifically in the DP. Note the anagen morphology of control and mutant follicles from P10 to P16. In contrast, while the mutant follicle at P18 (e) displays anagen morphology (a single follicle is shown), control follicles are in the early to mid catagen phase (different follicles at distinct stages are presented). Nuclei are in blue (DAPI). n>3 mice per genotype per time point. Scale bar, 25 µm. **(f)** Back skins were harvested and individually dissociated into a single-cell suspension. For each mouse skin, YFP-positive cells were sorted twice to obtain a purity of about 86% (right plot). The enrich mode was used for the first sort (left plot) and the purify mode was used for the second sort (middle). n=3 mice per genotype per stage. **(g)** Following sorting, total RNA was isolated and 100ng RNA was used to prepare a sequencing library for each mouse (n=3 mice per genotype per stage). Library samples, ran on a bioanalyzer, are shown. Note the fragment size for each library is 300-500bp.

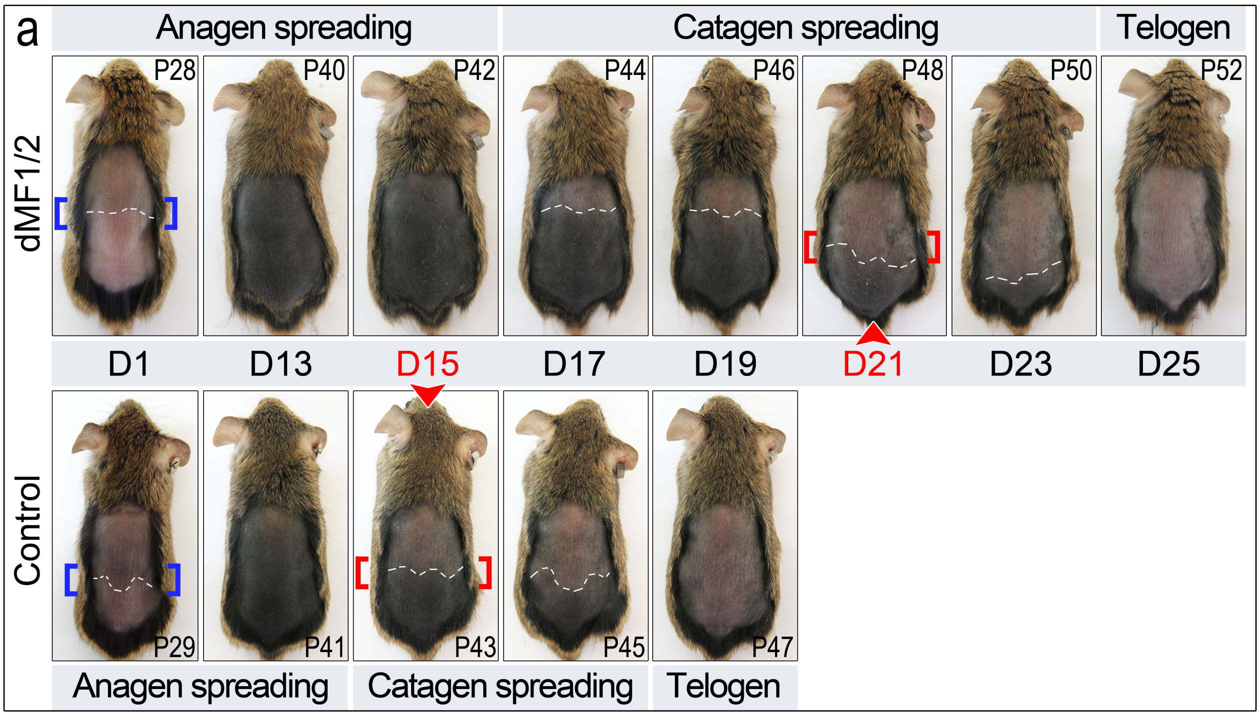

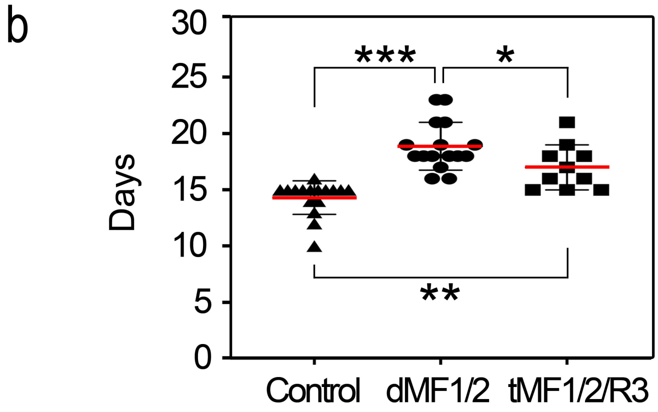

Supplementary Figure 4. Extended anagen of the second cycle in the dMF1/2 mutant partially depends on *Rspo3*. (a) Methodology used to estimate the duration of the anagen phase during the second cycle. The hair coat was clipped during the telogen of the first cycle when the skin is unpigmented. Images of the same dMF1/2 mutant (upper panels) and control (lower panels) that were taken at different times during the second hair cycle are displayed. The mouse age when the image was taken is indicated on the upper right (dMF1/2) or lower right (control). Pigmented skin is a clear indication for anagen that spreads from anterior to posterior. The border that distinguishes between pigmented and unpigmented skin defines the anagen wave front (dashed white line). Day 1 (D1) was defined when the anagen wave front is localized within the middle domain along the anterior-posterior axis (blue brackets). During anagen, the hair coat was repeatedly clipped to detect skin pigmentation and the catagen wave front that distinguishes between unpigmented and pigmented skin (dashed white line). Once the catagen wave front was found within the middle region along the anterior-posterior axis (red brackets), the anagen phase of the second cycle was defined as complete. Note that catagen spreading in the dMF1/2 mutant lasts longer than that of the control. **(b)** Anagen duration of the second cycle was quantified for controls (n=16), dMF1/2 double mutants (n=16) and tMF1/2/R3 triple mutants that concomitantly lack *Fgfr1*, *Fgfr2* and *Rspo3* (n=10). Note that anagen of the second cycle is significantly extended in the dMF1/2 double mutant and this extension partially requires the presence of *Rspo*3. Data are Mean±SD and presented by scatter dot plot. Two-tailed Mann-Whitney test was used: * P=0.032, ** P=0.001, *** P< 0.0001.

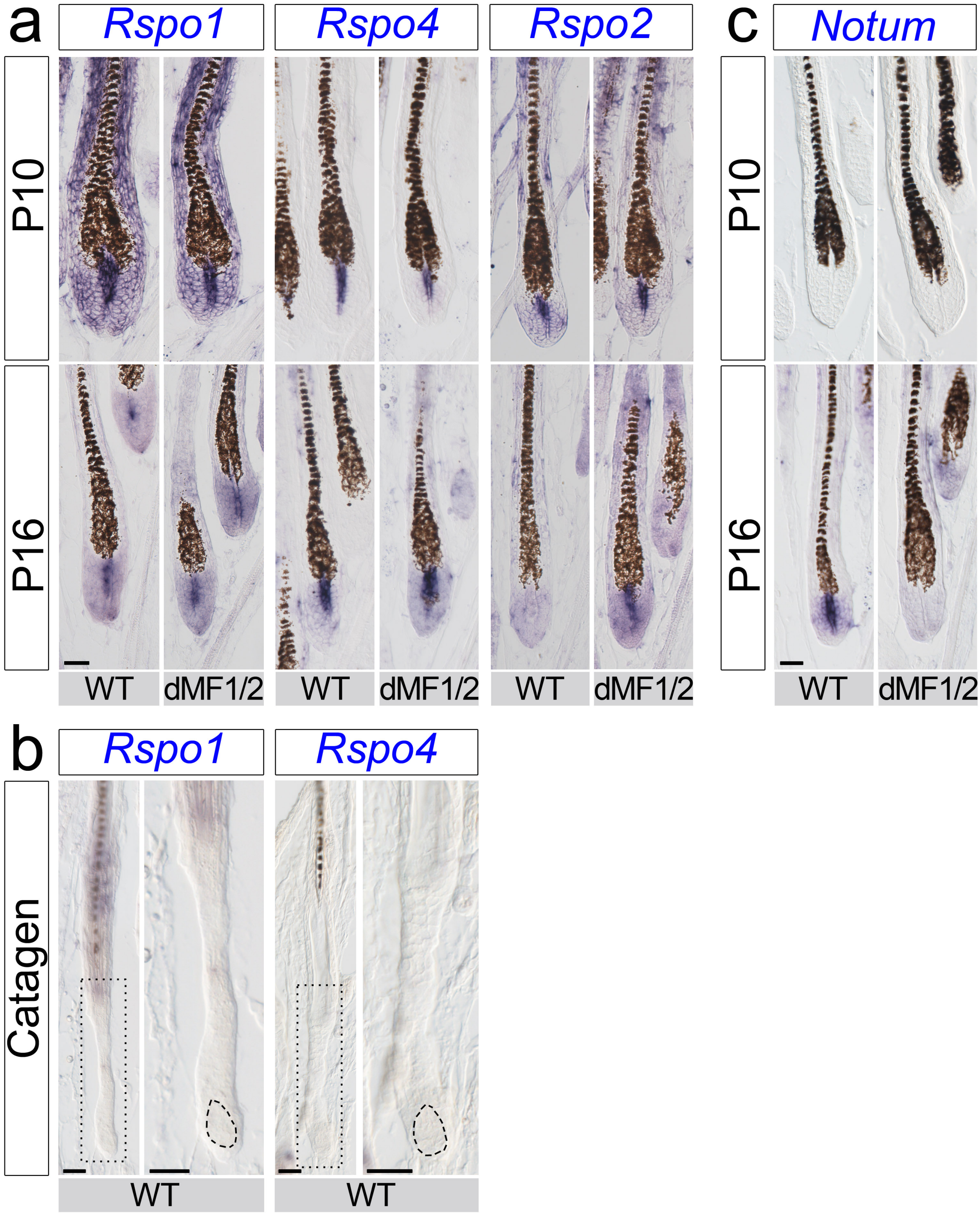

Supplementary Figure 5. Expression of Wnt agonists and antagonists in the DP during anagen is cooperatively altered to reduce Wnt activity in the matrix. (a) In-situ hybridization for *Rspo1*, *Rspo2* and *Rspo4* at P10 (upper panels) and P16 (lower panels) is shown to illustrate the predominant expression of these genes in the DP and their dynamic expression during mid to late anagen. **(b)** In-situ hybridization for *Rspo1* and *Rspo4* during catagen in wild type mice is displayed to illustrate their extinguished expression during this phase. On the right, higher magnifications of the fields in the dotted rectangles are shown. Dashed line demarcates the DP. **(c)** In-situ hybridization for *Notum* is shown to illustrate its upregulation in the DP of control mice during late anagen. RNA transcripts are in dark blue and pigmented hair shafts in black. n>3 mice per genotype per stage. Scale bar, 25 μm

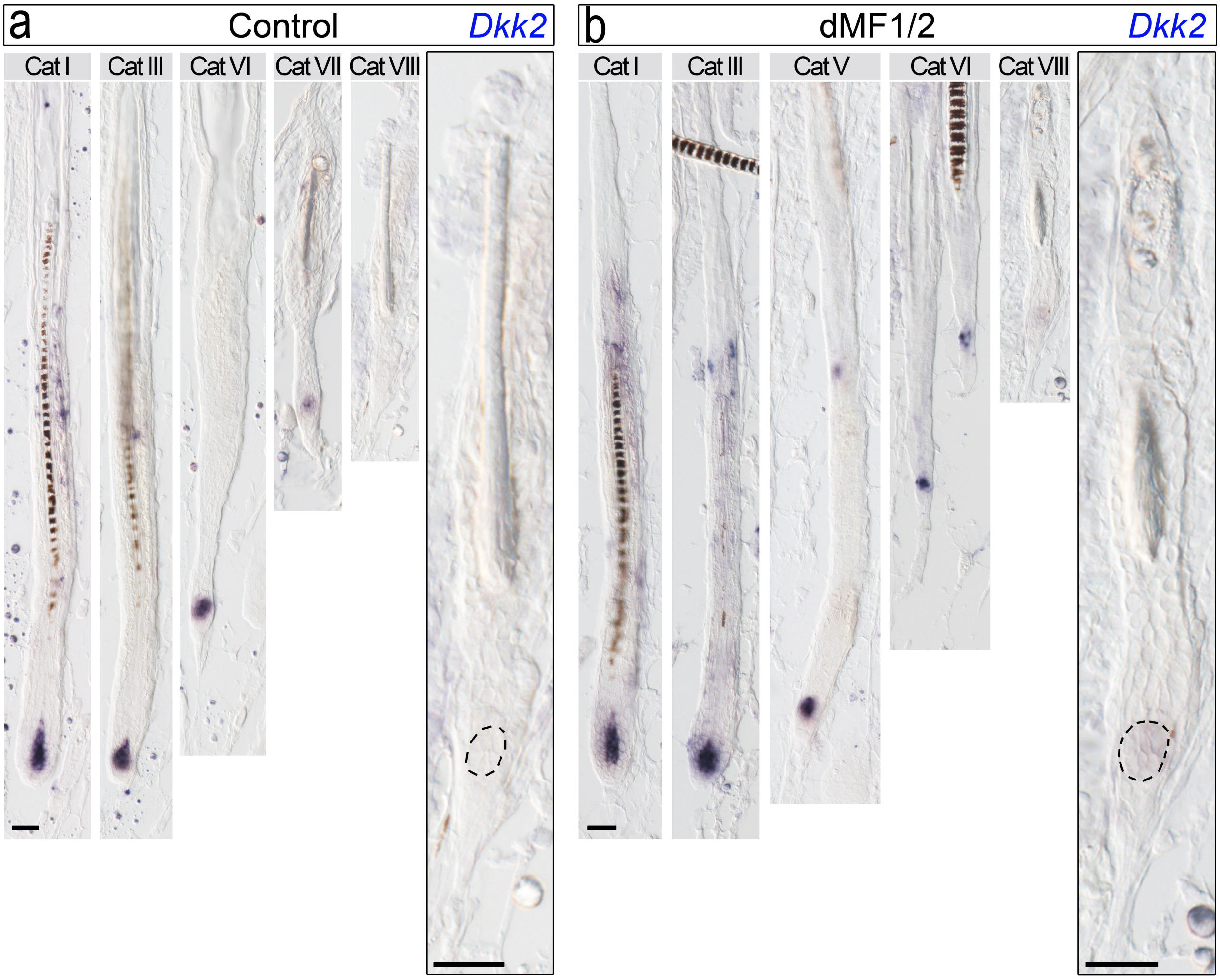

Supplementary Figure 6. *Dkk2* expression during catagen. In-situ hybridization for *Dkk2* during catagen in control (a) and dMF1/2 mutant (b) mice is shown. Single follicles at different stages of catagen are displayed and ordered chronologically from left to right. *Dkk2* transcripts are in blue and the specific catagen stage is indicated above the image. *Dkk2* expression is largely restricted to the DP during catagen and drops to undetectable levels towards the end of catagen in both control and dMF1/2 mutant mice. The rightmost panel (framed by the black rectangle) in both a and b is a higher magnification of the adjacent panel on the left to demonstrate the absence of *Dkk2* transcripts in the DP (demarcated by the black dashed line) at this late stage of catagen. n>3 mice per genotype per stage. Scale bar, 25 μm

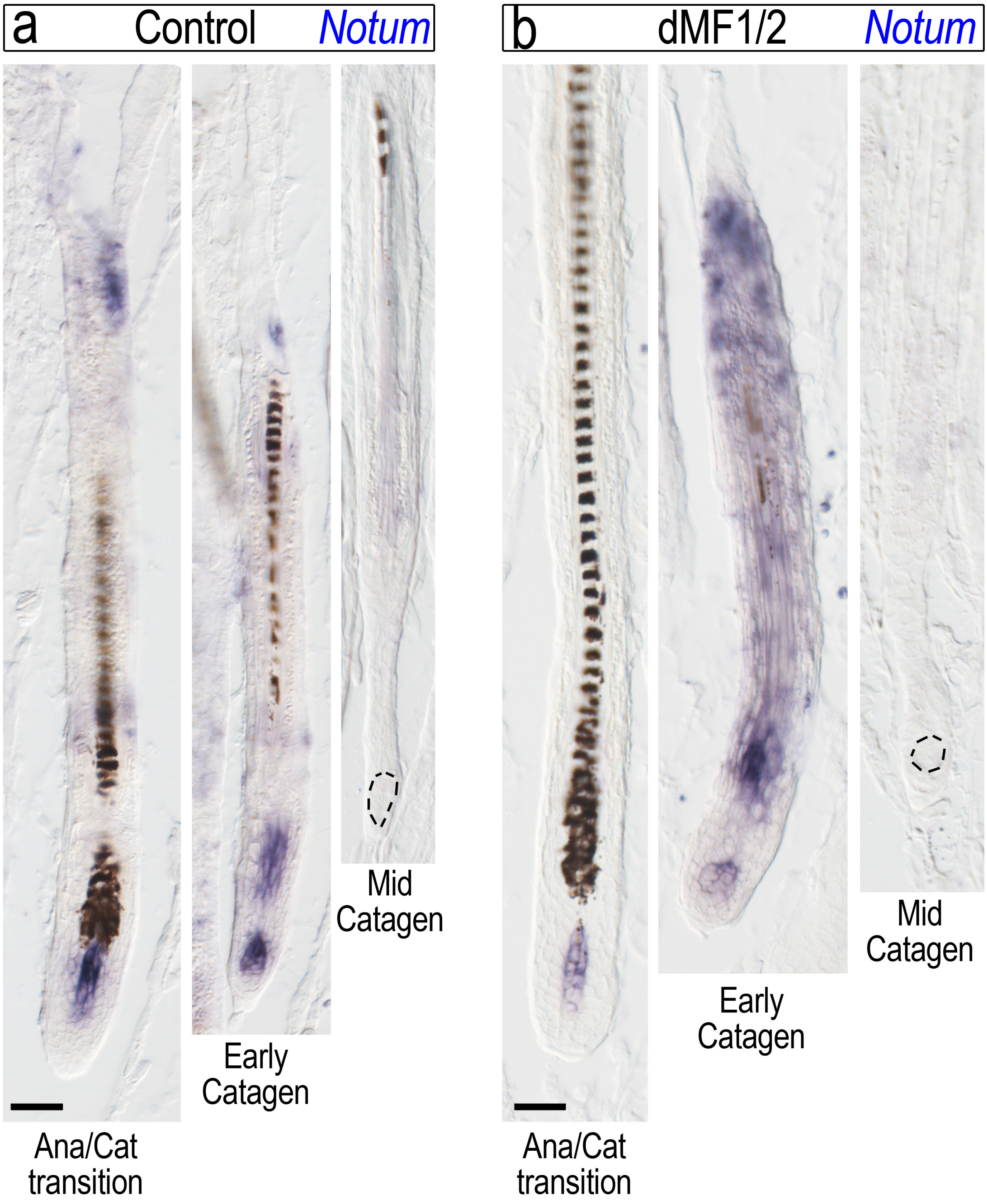

Supplementary Figure 7. *Notum* expression during catagen. In-situ hybridization for *Notum* during catagen in control (a) and dMF1/2 mutant (b) mice is shown. Follicles at the transition from anagen to catagen (left panel), early catagen (middle panel) and mid catagen (right panel) are depicted. Note that during catagen, *Notum* is also dynamically expressed in the regressing epithelial strand. *Notum* expression in the DP drops during mid catagen to undetectable levels (the DP is demarcated by the dashed line on the right panel). Also note the levels of *Notum* in the dMF1/2 mutant in the DP are lower than the control. n>3 mice per genotype per stage. Scale bar, 25 μm

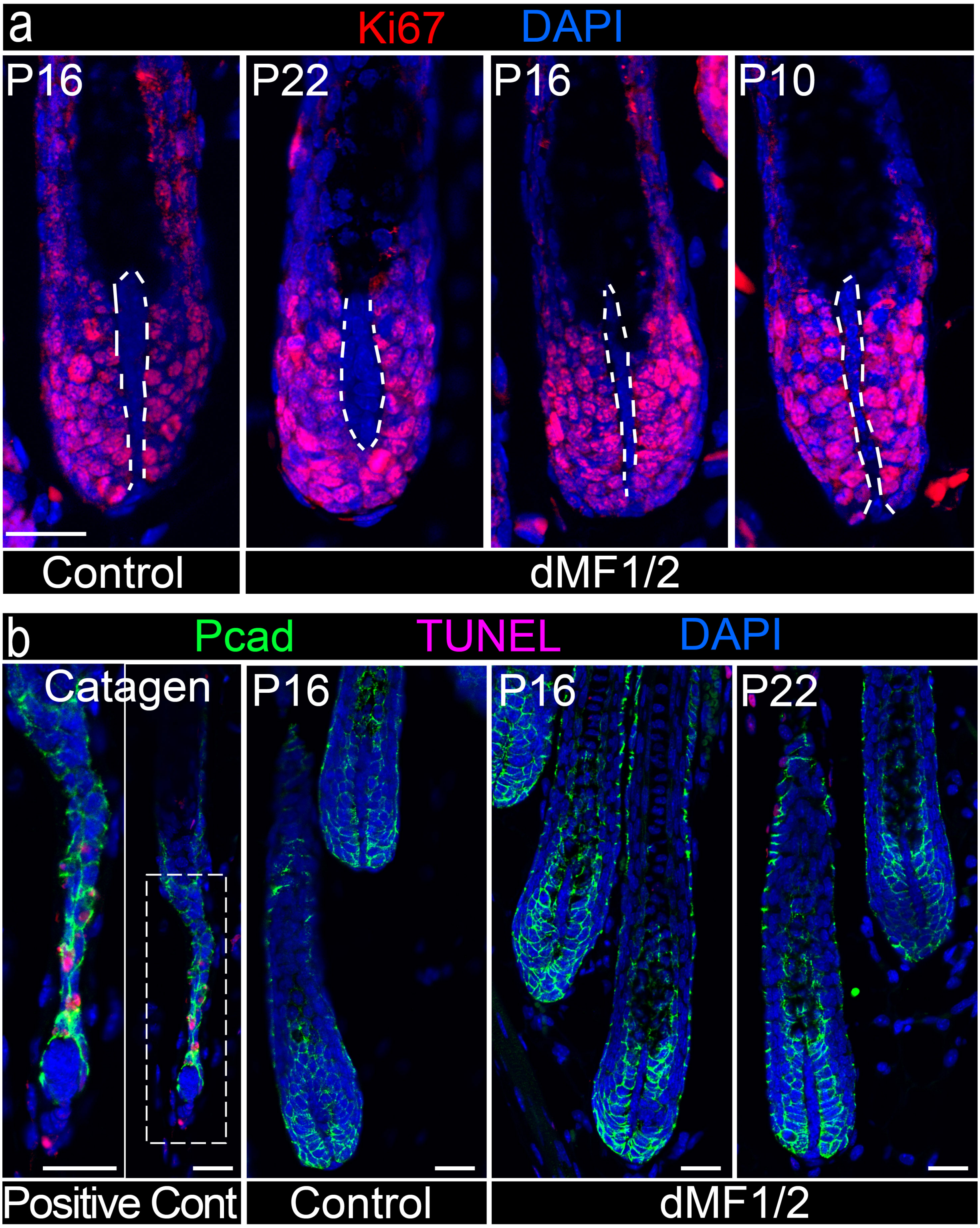

Supplementary Figure 8. The proliferative and survival state of the matrix during the late anagen of the dMF1/2 mutant is comparable to that of the late anagen of the control. (a) Ki67 immunostaining (red). On the leftmost panel, a control follicle at P16 is shown. On the right, dMF1/2 mutant follicles at P10, P16 and P22 are depicted. Note that P16 in the control and P22 in the dMF1/2 mutant represent late anagen. White dashed line demarcates the DP. Nuclei are in blue (DAPI). **(b)** Tunel staining (red). Note that immunostaining for Pcad (green) was also included to label matrix cells adjacent to the DP. A wild type follicle in catagen as a positive control for the Tunel staining is shown on the left and a higher magnification of the field within the dashed white rectangle is also displayed on the left panel. Note that apoptosis was not observed neither in control at P16 nor in dMF1/2 mutant at P16 or P22. n>3 mice per genotype per stage. Scale bar, 25μm.

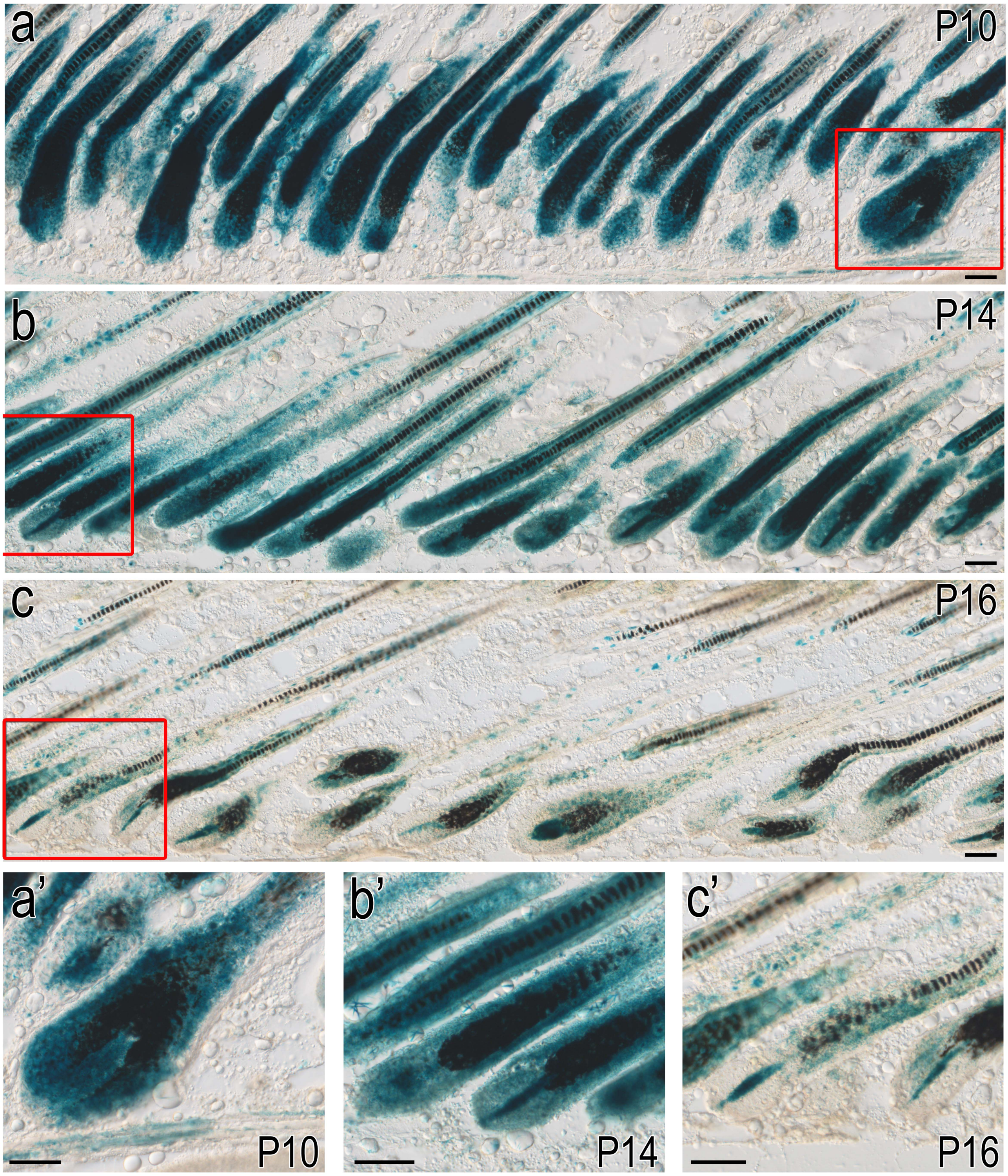

Supplementary Figure 9. The dynamics of Wnt activity levels in the matrix during anagen progression inversely differ than that in the DP. *Axin2-lacZ* reporter allele was used to detect Wnt activity. **(a-c)** LacZ staining of wild type mice at P10, P14 and P16 are depicted to demonstrate that Wnt activity during mid to late anagen progressively decreases in the matrix while increases in the DP. **(a'-c')** Higher magnifications of the fields within the red squares in a-c are shown respectively. n>3 mice per stage. Scale bar, 50μm.

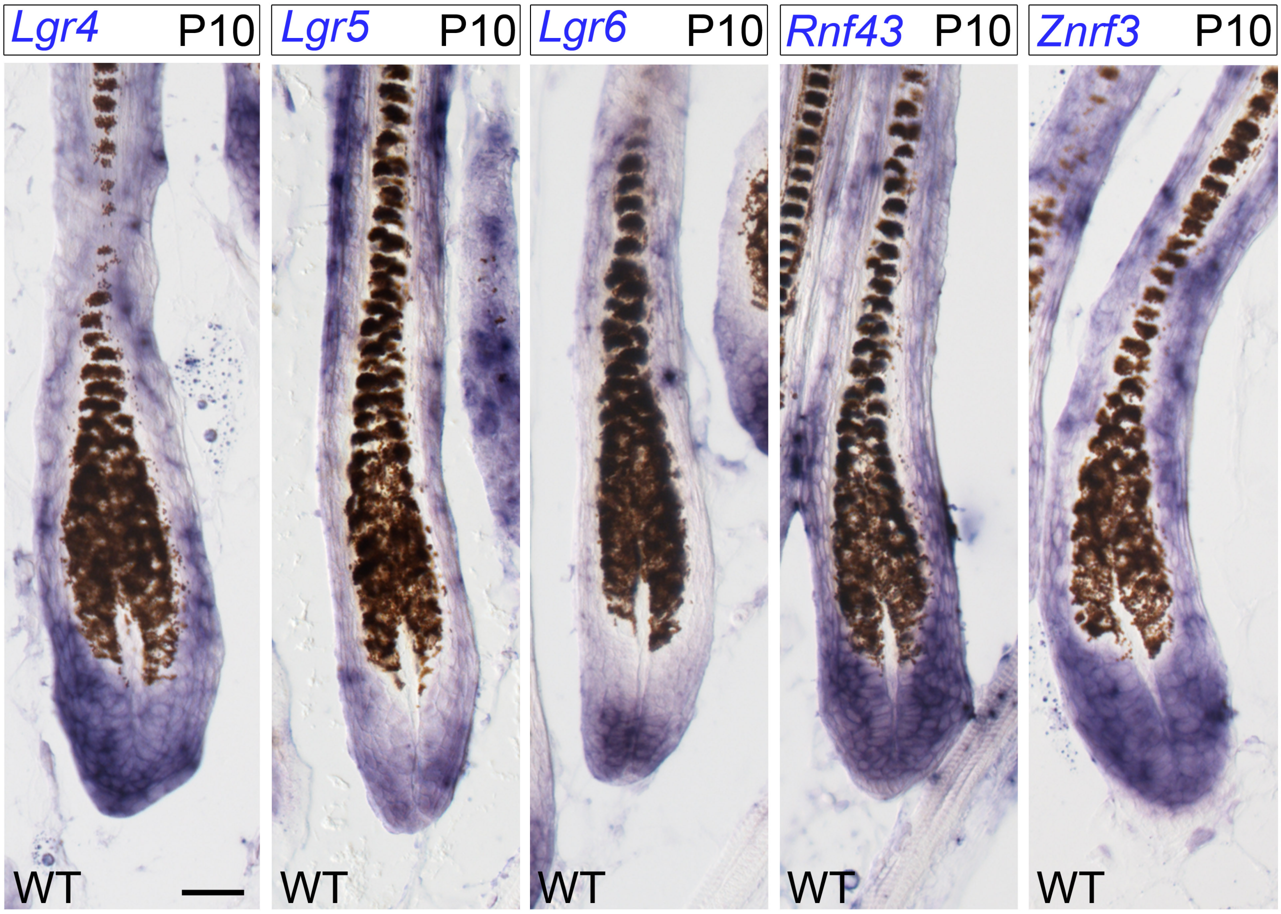

Supplementary Figure 10. Matrix cells are equipped with the molecular machinery that transduces Rspondins activity. In-situ hybridization for *Lgr4*, *Lgr5*, *Lgr6*, *Rnf43* and *Znrf3* detect abundant RNA transcripts (blue) in the matrix but not in the DP of wild type follicles at P10. Note the expression pattern of these genes at the RNA level resembles their expression at the protein level (compare with the immunostaining in Fig. 4e of main text). n>3 mice. Scale bar, 25μm.

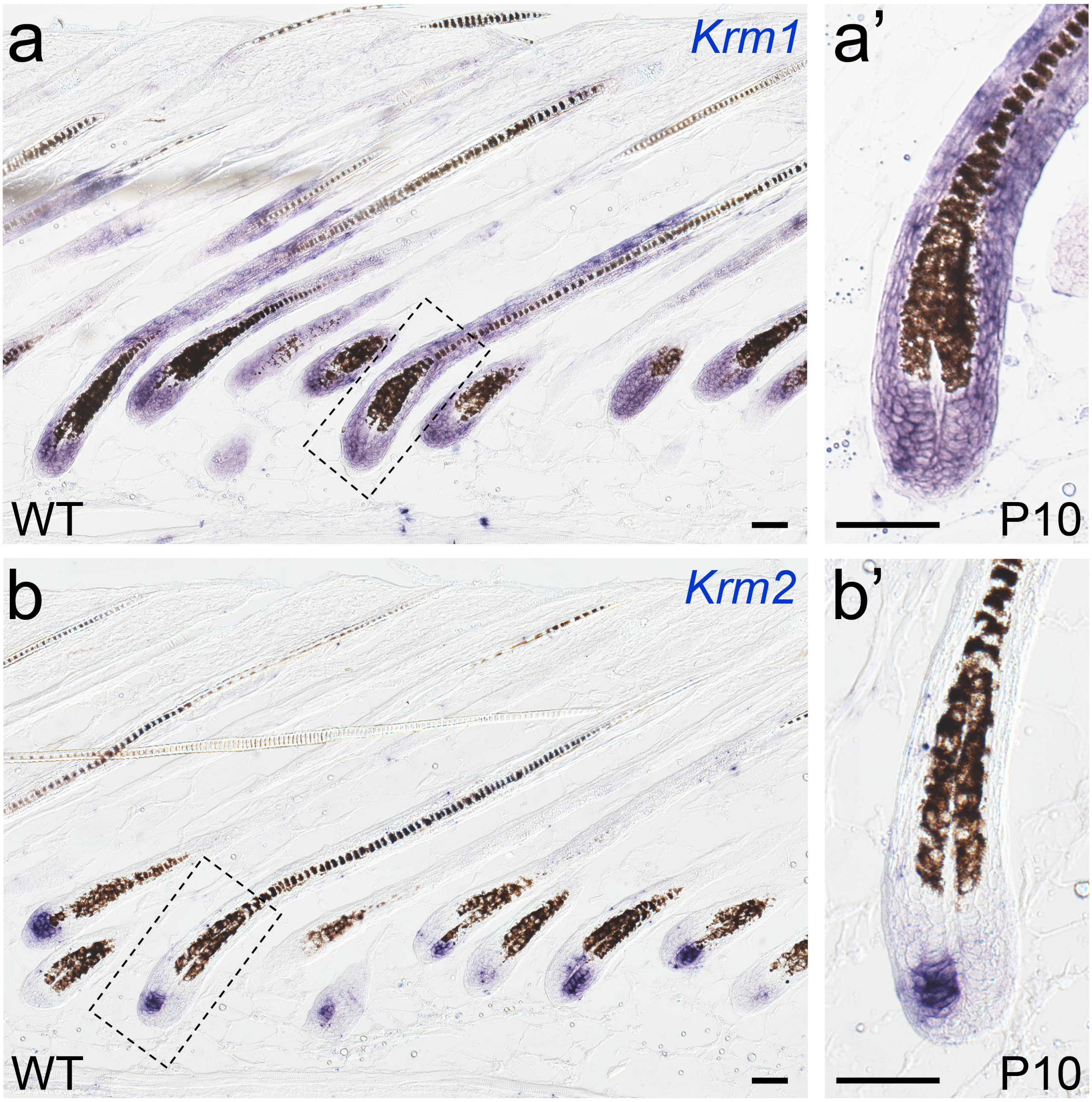

Supplementary Figure 11. Expression of *Krm1* and *Krm2* is restricted to the epithelial part of the follicle. (a) In-situ hybridization for *Krm1* in wild type mice is shown. **(a')** A higher magnification of a single follicle, outlined with the dashed square in a, is displayed to illustrate the lack of detectable expression of *Krm1* in wild type DP. **(b)** In situ hybridization for *Krm2* in wild type mice is presented. **(b')** A higher magnification of a single follicle, outlined with the dashed square in b, is shown to illustrate the restricted and asymmetrical expression of *Krm2* to only a small subgroup of matrix cells. RNA transcripts are in dark blue and pigmented hair shafts in black. n=3 mice. Scale bar, 50μm.

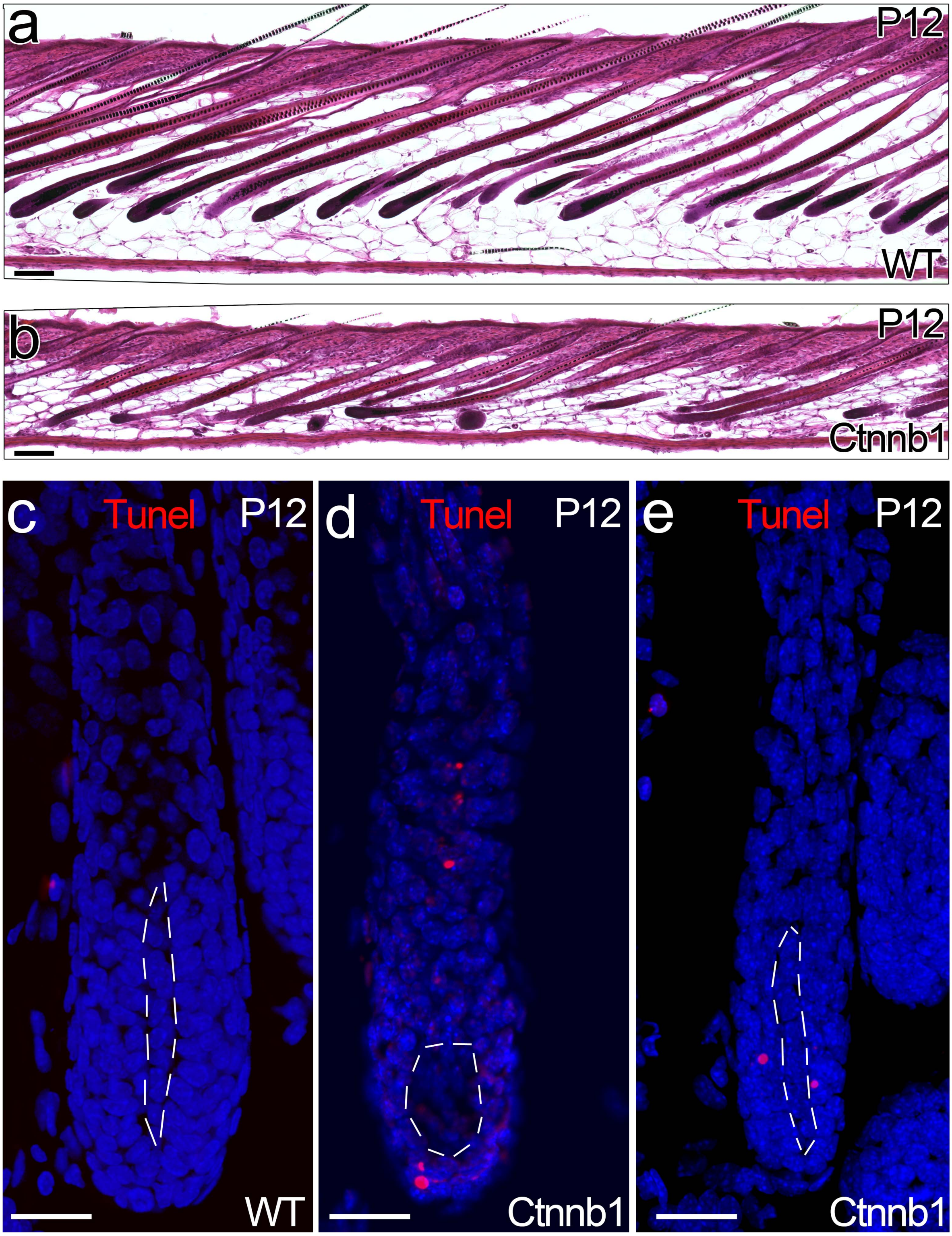

Supplementary Figure 12. Ablation of beta-catenin specifically in the DP results in premature induction of catagen. (a, b) HE staining of skin sections from wild type (a) and Ctnnb1 mutant (b) littermates at P12 is shown to demonstrate that while wild type follicles are still in anagen, mutant follicles have already entered catagen. n>3 mice per genotype **(c-e)** Tunel staining (red) of wild type and mutant follicles is shown to illustrate that apoptosis within the matrix is observed only in mutant follicles. Nuclei are in blue (DAPI). Dashed white lines outline the DP. Note that in the wild type follicle (c), no Tunel-positive cells were observed. Two mutant follicles at distinct stages of early catagen are displayed (d, e). Note the Tunel-positive cells around the DP. n>3 mice per genotype. Scale bar in a-b, 100μm. Scale bar in c-e, 25μm.

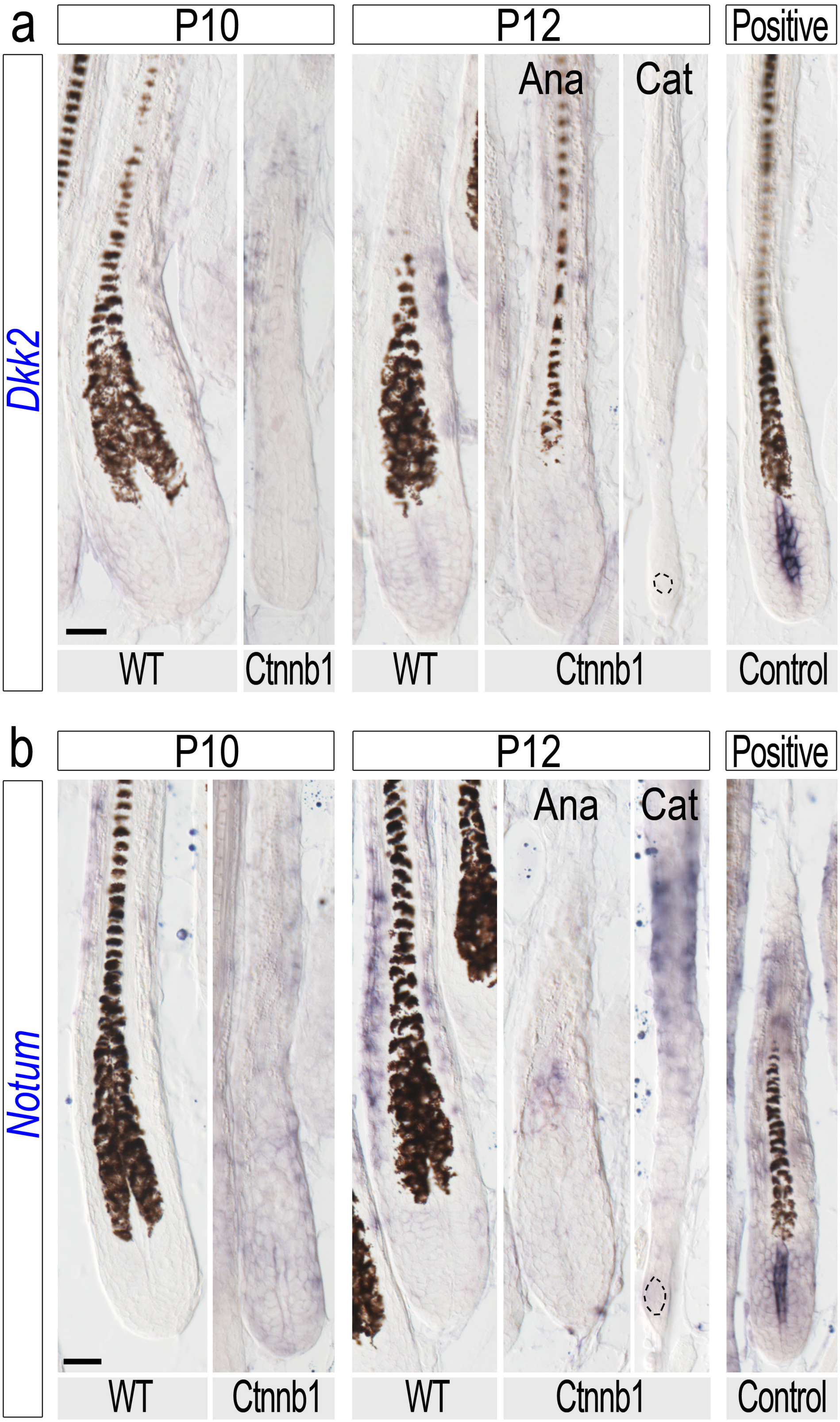

Supplementary Figure 13. Expression of *Dkk2* and *Notum* during the transition from anagen to catagen is not upregulated in the absence of beta-catenin in the DP. In situ hybridization for *Dkk2* (a) and *Notum* (b) is shown. Catagen induction in the beta-catenin mutant (Ctnnb1) occurs at about P12 and is unsynchronized. At P12, two Ctnnb1 mutant follicles are shown; on the left, a follicle with anagen morphology (Ana) is displayed, and on the right, a catagen follicle (Cat) from adjacent region is presented (dashed line demarcates the DP). On the rightmost panel, a positive control at late anagen when *Dkk2* and *Notum* are upregulated is included to normalize the detection period. n>3 mice per genotype per stage. Scale bar, 25μm.

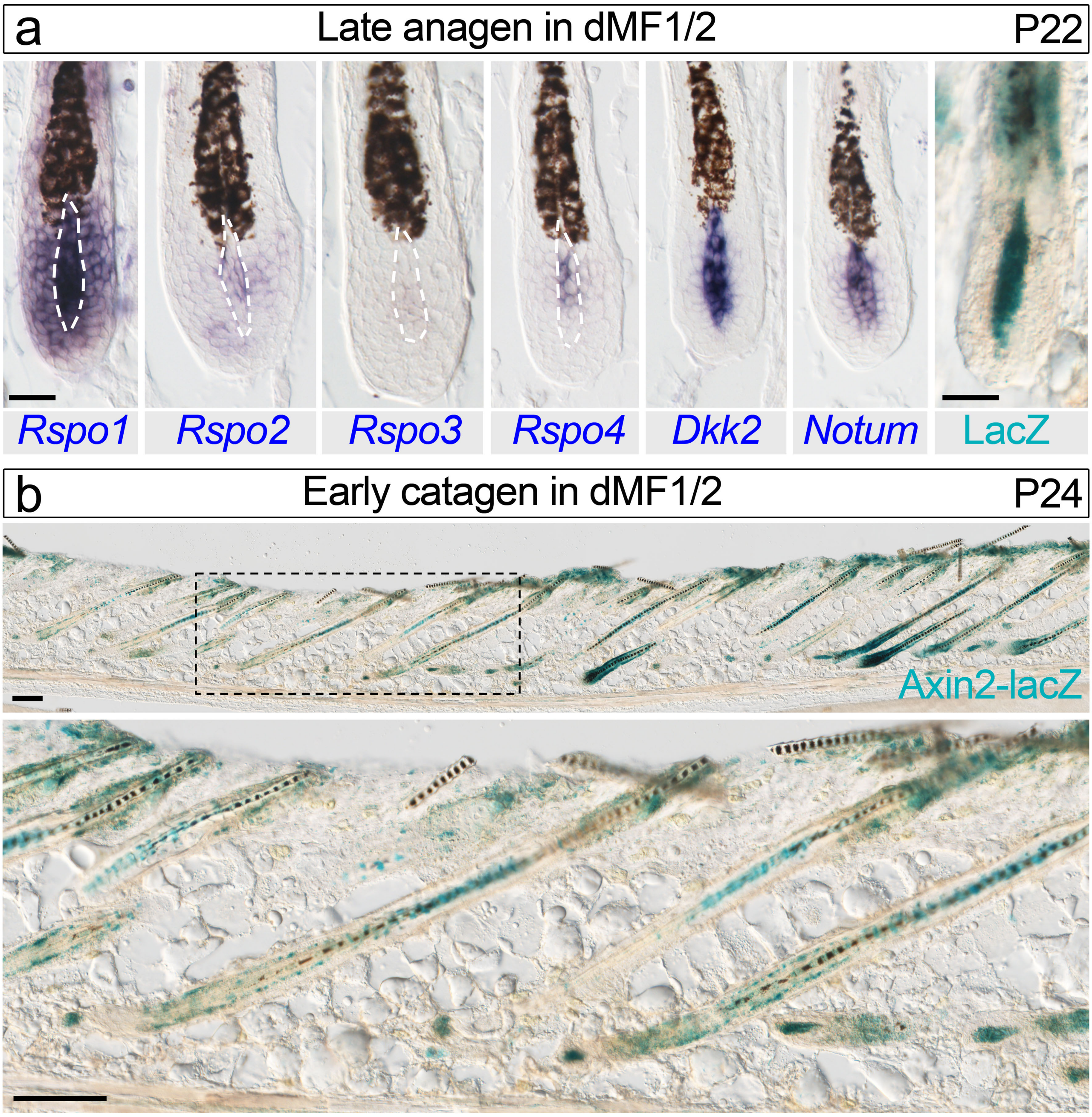

Supplementary Figure 14. The transition from anagen to catagen in the dMF1/2 mutant is molecularly normal. (a) In situ hybridization (blue) for Rspondins, *Dkk2* and *Notum* at P22 of dMF1/2 mutant are shown. On the rightmost panel, LacZ staining at P22 is shown to illustrate the persistence and absence of Wnt activity in the DP and matrix, respectively. The dashed white line demarcates the DP. n>3 mice. Scale bar, 25μm. **(b)** LacZ staining of dMF1/2 mutant at P24 during early catagen is shown to demonstrate the presence of Wnt activity in the DP and the absence of Wnt activity around the DP. A higher magnification of the field within the dashed black rectangle in the upper panel is displayed in the lower panel. n=3 mice. Scale bar, 100μm.

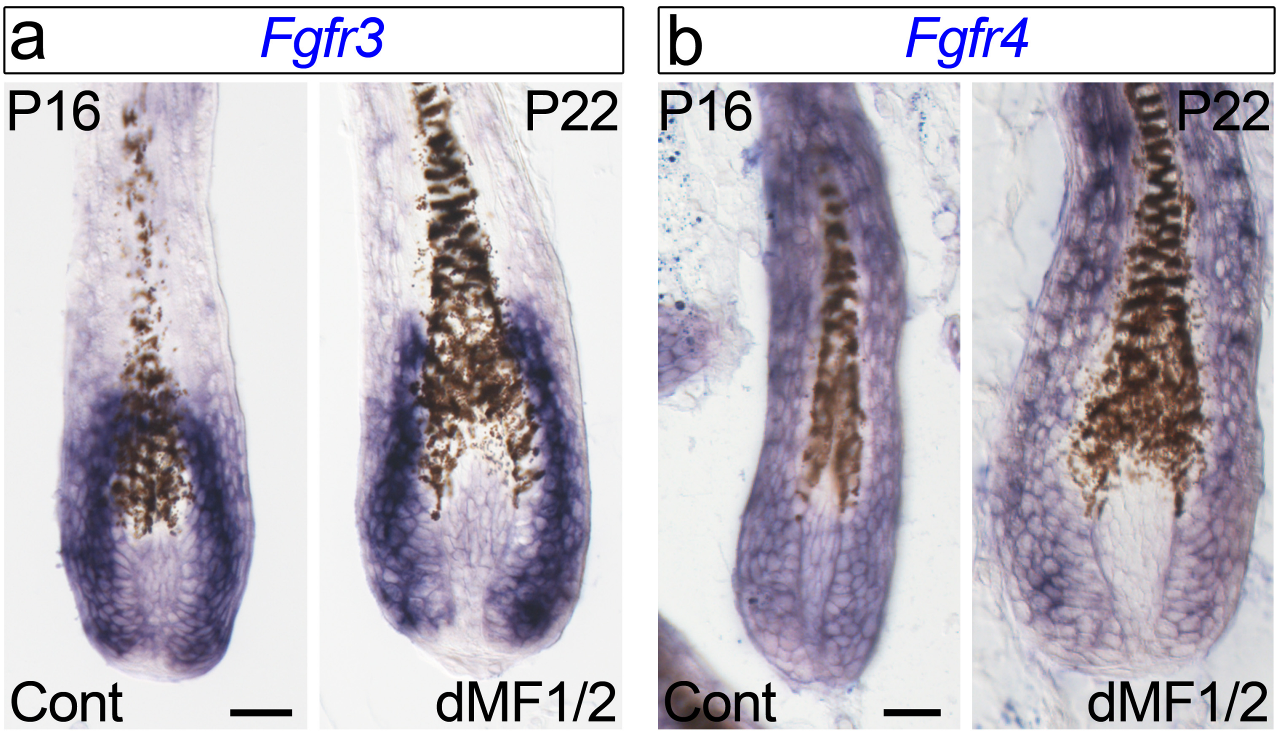

Supplementary Figure 15. *Fgfr3* and *Fgfr4* expression in the DP is not upregulated in the dMF1/2 mutant. In-situ hybridization for *Fgfr3* (a) and *Fgfr4* (b) is shown to compare between age-unmatched but stage-matched (late anagen) control (P16) and dMF1/2 (P22) mutant mice. n>3 mice per genotype per stage. **(a)** Note the low and unaltered levels of *Fgfr3* transcripts (blue) in the DP of control and dMF1/2 mutant follicles. **(b)** Note the low levels of *Fgfr4* transcripts (blue) in the DP of control follicle and the reduced levels in the DP of dMF1/2 mutant follicle. Scale bar, 25μm.
