# Supplementary material for: Fgf and Wnt signaling interaction in the mesenchymal niche regulates the murine hair cycle clock
Source: Nat Commun. 2020 Oct 9;11:5114. doi: 10.1038/s41467-020-18643-x (PMC7547083; doi:10.1038/s41467-020-18643-x)
Supplement: Supplementary file 2 — Reporting Summary [file 41467_2020_18643_MOESM2_ESM.pdf]

nature research

Corresponding author(s): David Enshell-Seijffers

Last updated by author(s): Aug 9, 2020

Reporting Summary

Nature Research wishes to improve the reproducibility of the work that we publish. This form provides structure for consistency and transparency in reporting. For further information on Nature Research policies, see our Editorial Policies and the Editorial Policy Checklist.

Statistics

For all statistical analyses, confirm that the following items are present in the figure legend, table legend, main text, or Methods section.

n/a	Confirmed	
☐	☒	The exact sample size (*n*) for each experimental group/condition, given as a discrete number and unit of measurement
☐	☒	A statement on whether measurements were taken from distinct samples or whether the same sample was measured repeatedly
☐	☒	The statistical test(s) used AND whether they are one- or two-sided<br>*Only common tests should be described solely by name; describe more complex techniques in the Methods section.*
☐	☒	A description of all covariates tested
☐	☒	A description of any assumptions or corrections, such as tests of normality and adjustment for multiple comparisons
☐	☐	A full description of the statistical parameters including central tendency (e.g. means) or other basic estimates (e.g. regression coefficient) AND variation (e.g. standard deviation) or associated estimates of uncertainty (e.g. confidence intervals)
☐	☒	For null hypothesis testing, the test statistic (e.g. *F*, *t*, *r*) with confidence intervals, effect sizes, degrees of freedom and *P* value noted<br>*Give P values as exact values whenever suitable.*
☒	☐	For Bayesian analysis, information on the choice of priors and Markov chain Monte Carlo settings
☒	☐	For hierarchical and complex designs, identification of the appropriate level for tests and full reporting of outcomes
☒	☐	Estimates of effect sizes (e.g. Cohen's *d*, Pearson's *r*), indicating how they were calculated

Our web collection on statistics for biologists contains articles on many of the points above.

Software and code

Policy information about availability of computer code

Data collection	Microscopy Images were acquired using Zen Black 11 (service pack 7) and Zen Blue 2.3 software, and finalized using Photoshop CS5.1. FACS data was obtained with Summit program version 6.3.1.
Data analysis	RNASeq data Bioinformatic analysis was performed with Trimmomatic (Version 0.27) (Bolger et al., 2014), Tophat (version 2) (Kim et al., 2013), HTSeqcount tool (Version 0.8) (Anders et al., 2015), DESeq2 (Version 1.16) (Love et al., 2014).

For manuscripts utilizing custom algorithms or software that are central to the research but not yet described in published literature, software must be made available to editors and reviewers. We strongly encourage code deposition in a community repository (e.g. GitHub). See the Nature Research guidelines for submitting code & software for further information.

Data

Policy information about availability of data

All manuscripts must include a data availability statement. This statement should provide the following information, where applicable:

- Accession codes, unique identifiers, or web links for publicly available datasets
- A list of figures that have associated raw data
- A description of any restrictions on data availability

Upon request, all the data that support the results of this study are available from the corresponding author.

Field-specific reporting

Please select the one below that is the best fit for your research. If you are not sure, read the appropriate sections before making your selection.

☒ Life sciences ☐ Behavioural & social sciences ☐ Ecological, evolutionary & environmental sciences

For a reference copy of the document with all sections, see nature.com/documents/nr-reporting-summary-flat.pdf

Life sciences study design

All studies must disclose on these points even when the disclosure is negative.

Sample size	The sample sizes were chosen based on previous studies with similar methodologies (Avigad-Laron el al. 2018 Cell Reports).
Data exclusions	No data were excluded from the analysis.
Replication	All attempts at replication were successful and at least 3 replicates were performed.
Randomization	Wild type and mutant mice were selected based on their genotype.
Blinding	Blinding was not used since control and mutant mice are readily distinguished based on their phenotypes.

Reporting for specific materials, systems and methods

We require information from authors about some types of materials, experimental systems and methods used in many studies. Here, indicate whether each material, system or method listed is relevant to your study. If you are not sure if a list item applies to your research, read the appropriate section before selecting a response.

Materials & experimental systems

n/a	Involved in the study	
☐	☒	Antibodies
☒	☐	Eukaryotic cell lines
☒	☐	Palaeontology and archaeology
☐	☒	Animals and other organisms
☒	☐	Human research participants
☒	☐	Clinical data
☒	☐	Dual use research of concern

Methods

n/a	Involved in the study	
☒	☐	ChIP-seq
☐	☒	Flow cytometry
☒	☐	MRI-based neuroimaging

Antibodies

Antibodies used	Lgr4 (Rabbit polyclonal to GPCR GPR48 - C-terminal, 1:200, Abcam Ab224480) Lgr5(Rabbit monoclonal [EPR3065Y] to LGR5, 1:200, Abcam Ab75850), Lgr6 (Rabbit Polyclonal to GPCR LGR6, 1:200, Abcam Ab214325), Rnf43(Rabbit polyclonal to RNF43, 1:200, Abcam Ab217787), Znrf3(Rabbit polyclonal,1:200, Bioss Abs, bs-9141-R), Bcat(Mouse monoclonal [15B8] to beta Catenin, 1:200, Abcam Ab6301), P-cadherin (Rat monoclonal anti P-Cadherin, clone 106020, R&D SYSTEMS #MAB761 1:100), Ki67 (Rabbit polyclonal to Ki67, Abcam Ab15580, 1:100).
Validation	Antibody validation information can be found on manufacturers' website.<br>Anti Lgr4 (https://www.abcam.com/gpcr-gpr48-antibody-c-terminal-ab224480.html), Anti Lgr5 (https://www.abcam.com/lgr5-antibody-epr3065y-ab75850.html) , Anti Lgr6 (https://www.abcam.com/gpcr-lgr6-antibody-ab214325.html), Anti Rnf43 (https://www.abcam.com/rnf43-antibody-ab217787.html) , Anti Beta-catenin (https://www.abcam.com/beta-catenin-antibody-15b8-ab6301.html) , Anti Znrf3 (https://www.biossusa.com/products/bs-9141r), Anti Ki67 (https://www.abcam.com/ki67-antibody-sp6-ab16667.html), Anti P-cadherin (https://www.rndsystems.com/products/human-mouse-p-cadherin-antibody-106020_mab761)

Animals and other organisms

Policy information about studies involving animals; ARRIVE guidelines recommended for reporting animal research

Laboratory animals	Fgfr1Flox/Flox (Fgfr1tm5Sor)(Hoch et al., 2006), Fgfr2Flox/Flox (Fgfr2tm1Dor)(Yu et al., 2003), Ctnnb1Flox/Flox (Ctnnb1tm2Kem) (Brault et al, 2001), ROSA26 YFP reporter (Gt(ROSA)26Sortm1(EYFP)Cos)(Srinivas et al., 2001), and Axin2-lacZ reporter (Axin2tm1Wbm) (Lustig et al., 2002), were obtained from Jackson Lab. The DP-specific Corin-cre (Corintm2Bamo) mouse was provided by Bruce Morgan (Harvard medical school). Rspo3Flox/Flox mice were kindly provided by Chritof Niehrs (Kazanskaya, 2008). Mice were bred to a mixed background and collected between postnatal<br>day 8 to 28. Both genders were used.

Wild animals	The study did not involve wild animals.
Field-collected samples	The study did not involve samples collected from the field.
Ethics oversight	The Institutional Animal Care and Use Committee of Bar Ilan University

Note that full information on the approval of the study protocol must also be provided in the manuscript.

Flow Cytometry

Plots

Confirm that:

- [x] The axis labels state the marker and fluorochrome used (e.g. CD4-FITC).
- [x] The axis scales are clearly visible. Include numbers along axes only for bottom left plot of group (a 'group' is an analysis of identical markers).
- [x] All plots are contour plots with outliers or pseudocolor plots.
- [x] A numerical value for number of cells or percentage (with statistics) is provided.

Methodology

Sample preparation	To obtain a single-cell suspension, whole skin was placed dermis side down in 0.25% Trypsin (GIBCO) at 4°C overnight, minced and stirred for 1hr in 0.2% collagenase at 37°C. The cells were then filtered with strainers (100µM, 70µM, and 40µM).
Instrument	MoFlo Astrios (Backman Coulter)
Software	Summit program
Cell population abundance	Pre-Enrichment target cell population with an average abundance of 1.13%, post- enrichment 62%. Sample purity was determined by analysis of yfp% from final sample (86%).
Gating strategy	DP cell labelled endogenously with YFP were purified through a two-step protocol. Step 1 (Enrichment) was performed on 1-2 mode collecting YFP% positive cells, then Step 2 (Purify) was performed on 1-mode.

- [x] Tick this box to confirm that a figure exemplifying the gating strategy is provided in the Supplementary Information.
